# Hierarchical assembly of centriole subdistal appendages via centrosome binding proteins CCDC120 and CCDC68

**DOI:** 10.1038/ncomms15057

**Published:** 2017-04-19

**Authors:** Ning Huang, Yuqing Xia, Donghui Zhang, Song Wang, Yitian Bao, Runsheng He, Junlin Teng, Jianguo Chen

**Affiliations:** 1 Key Laboratory of Cell Proliferation and Differentiation of the Ministry of Education, College of Life Sciences, Peking University, Beijing 100871, China; 2 State Key Laboratory of Membrane Biology, College of Life Sciences, Peking University, Beijing 100871, China; 3 Center for Quantitative Biology, Peking University, Beijing 100871, China

## Abstract

In animal cells, the centrosome is the main microtubule-organizing centre where microtubules are nucleated and anchored. The centriole subdistal appendages (SDAs) are the key structures that anchor microtubules in interphase cells, but the composition and assembly mechanisms of SDAs are not well understood. Here, we reveal that centrosome-binding proteins, coiled-coil domain containing (CCDC) 120 and CCDC68 are two novel SDA components required for hierarchical SDA assembly in human cells. CCDC120 is anchored to SDAs by ODF2 and recruits CEP170 and Ninein to the centrosome through different coiled-coil domains at its N terminus. CCDC68 is a CEP170-interacting protein that competes with CCDC120 in recruiting CEP170 to SDAs. Furthermore, CCDC120 and CCDC68 are required for centrosome microtubule anchoring. Our findings elucidate the molecular basis for centriole SDA hierarchical assembly and microtubule anchoring in human interphase cells.

## Abstract (web summary)

Centriole subdistal appendages (SDAs) anchor microtubules in interphase cells, but their composition and assembly mechanisms are unclear. Here the authors show that two new SDA components, CCDC120 and CCDC68, are required for hierarchical SDA assembly and centrosome microtubule anchoring.

Microtubules play important roles in many cellular events, including vesicular trafficking, cell migration, polarization and division. Temporal and spatial regulation of microtubule dynamics are crucial for proper cellular function. The major microtubule-organizing centre in animal cells is the centrosome, which is surrounded by pericentriolar material and contains mother and daughter centrioles. In contrast with daughter centrioles, mother centrioles are characterized by two projection structures, the distal appendages (DAs) and subdistal appendages (SDAs), which are localized to the distal and subdistal ends, respectively^1^,^2^,^3^. The DAs mainly function in membrane docking and ciliogenesis^4^, while the SDAs anchor the microtubule minus-ends to the centrosome in interphase cells^5^,^6^.

Microtubule nucleation and anchoring at centrosomes are essential processes for microtubule organization in interphase cells^1^,^6^. Protein complexes that function in microtubule nucleation at centrosomes, such as the γ-tubulin ring complex, have been well studied^7^,^8^,^9^,^10^. In contrast, although some centrosome proteins, including Ninein^11^, ODF2 (also known as cenexin)^12^, CEP170 (ref. 13), CEP110 (also known as centriolin)^14^, CC2D2A (ref. 15) and ɛ-tubulin^16^, have been classified as SDA components by electron microscopy or three-dimensional structured illumination microscopy (3D-SIM)^17^, the composition and functions of the SDAs are just beginning to be revealed. Ninein acts as a microtubule-anchoring protein that recruits microtubule nucleation protein complex γ-tubulin ring complex via its N terminus and localizes to the centrosome via its C terminus in mouse cells^18^. CEP170 interacts with Ninein and associates with microtubules through its C terminus^13^,^19^. Unlike Ninein and CEP170, ODF2 is localized much closer to the ‘barrel' of the mother centriole and is critical for DA and SDA formation^20^. Recent studies have shown that ODF2 controls DA and SDA assembly through different domains^21^. Depleting these SDA proteins disturbs microtubule anchorage to the centrosomes in interphase cells^11^,^12^,^13^,^14^,^15^. Other proteins, including those comprising the dynein/dynactin complex (containing p50/dynamitin, p150^Glued^ and p24), EB1, Kif3a and trichoplein (TCHP), which localize near SDAs or the subdistal ends of centrioles, also function in anchoring microtubules to mother centrioles^22^,^23^,^24^,^25^,^26^,^27^. In addition to their functions in microtubule anchoring, proteins that localize to or near the SDAs also regulate endosome recycling^28^, spindle orientation^29^ and ciliogenesis^15^,^22^,^30^,^31^,^32^,^33^,^34^. Although recent studies have made progress in understanding the molecular basis of microtubule regulation of SDA components in interphase cells, the molecular connections and assembly order remain poorly understood.

Coiled-coil domain containing 120 (CCDC120) was first identified as a centrosome protein by a centrosome proteomics study^35^. CCDC120 interacts with cytohesin-2 to regulate vesicular trafficking and neurite growth^36^. Coiled-coil domain containing 68 (CCDC68) was recently reported as a tumour suppressor in a study of patients with pancreatic ductal adenocarcinoma^37^. However, the functions of CCDC120 and CCDC68 in the centrosome are unknown. In this study, we identify CCDC120 and CCDC68 as SDA components and propose a hierarchical assembly model of SDAs by uncovering their roles in cooperating with known SDA components such as ODF2, Ninein and CEP170, as well as in microtubule anchoring in interphase cells.

## Results

### CCDC120 is an SDA component

Human CCDC120 contains 630 amino acids and is predicted to contain two short coiled-coil domains in its N terminus and a long proline-rich domain through its C terminus (Supplementary Fig. 1a). To characterize CCDC120, we generated rabbit and mouse polyclonal antibodies against its N terminus (1–200 amino acids (aa), Supplementary Fig. 1a). Both antibodies recognized a band at ∼85 kDa by immunoblotting in U2OS cells, which was of similar size as that recognized by a commercial anti-CCDC120 antibody (generated against 281–630 aa), and nearly abolished after transfection with short interfering RNA (siRNA) targeting CCDC120 (Supplementary Fig. 1b,c), although a nonspecific band at ∼60 kDa was detected by the rabbit polyclonal antibody (Supplementary Fig. 1b). CCDC120 co-fractionated with centrosome components hNinein, CEP170, ODF2 and γ-tubulin (Supplementary Fig. 1d), and was localized at the centrosome through its two coiled-coil domain-containing regions (1–90 aa and 91–320 aa) during the cell cycle (Supplementary Fig. 1e–g), indicating that CCDC120 is a centrosome protein.

**Figure 1:**
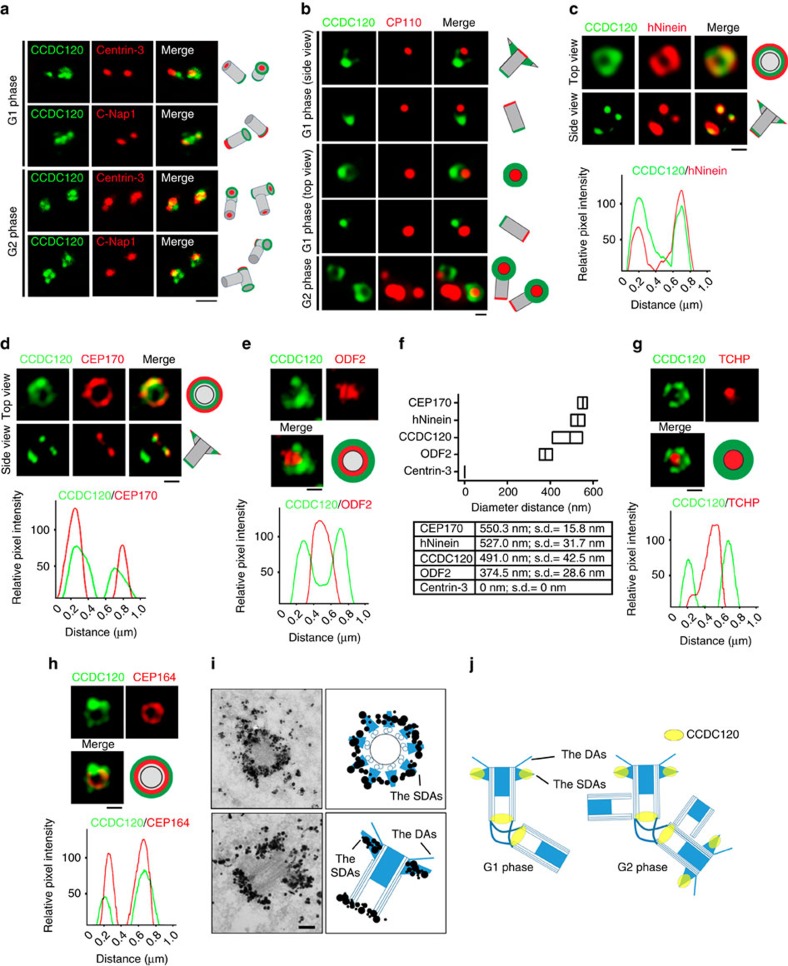
CCDC120 is localized at the centrosome. (**a**) Immunofluorescence of CCDC120 (green) and Centrin-3 (red), or C-Nap1 (red) in U2OS cells. Scale bar, 1 μm. (**b**–**e**) 3D-SIM images of U2OS cells double-immunostained with antibodies against CCDC120 (green) and CP110 (**b**, red), hNinein (**c**, red), CEP170 (**d**, red) or ODF2 (**e**, red). Scale bars, 500 nm. The intensity plots of the rings in **c**–**e** are, respectively, shown below. (**f**) Average diameter of the ring-like structure formed by listed proteins. The low-high bars (horizontal) show the range of the diameter and the vertical lines indicate the mean. From the bottom to the top: *n*=10; 15; 28; 19; and 24. (**g**,**h**) 3D-SIM images of U2OS cells double-immunostained with antibodies against CCDC120 (green) and TCHP (**g**, red) or CEP164 (**h**, red). Scale bars, 500 nm. The intensity plots of the rings are, respectively, shown below. (**i**) Immuno-EM images. U2OS cells were labelled with anti-CCDC120 antibody followed by nanogold-coupled secondary antibody. Schematics of immuno-EM images are shown. Scale bar, 200 nm. (**j**) Schematic of CCDC120 localization at centrosomes. CCDC120 localization is shown in yellow.

To investigate the localization of CCDC120 at the centrosome in detail, we performed immunostaining to detect CCDC120. Three CCDC120-positive dots were observed in the centrosome with both lab-generated and commercial antibodies (Fig. 1a and Supplementary Fig. 1h), of which one relatively poorly defined dot was partially co-localized with Centrin-3, a marker of the distal centriole ends, in one centriole, whereas the other two dots co-localized with C-Nap1, a marker of the proximal centrioles ends, in both centrioles during the G1 phase (Fig. 1a, top). This asymmetric CCDC120 localization changed as the cells entered the G2 phase, when CCDC120 was localized at the distal and proximal ends of the parental centrioles (Fig. 1a, bottom). To further assess CCDC120 localization at the distal end of the centriole, 3D-SIM was performed. Interestingly, we observed that CCDC120 appeared as two ‘dim dots' in the side views (Fig. 1b, top) or as a ‘ring-like' structure in the top views (Fig. 1b, central, and Supplementary Fig. 1i) at the distal part of one centriole in G1 phase cells; these structures were localized near CP110 and appeared at both parental centrioles in G2 phase cells (Fig. 1b, bottom).

Because the localization of CCDC120 is very similar to that of Ninein, a centrosome protein localized to the SDAs of mother centrioles and proximal ends of both centrioles^11^,^18^, we co-immunostained CCDC120 with human Ninein (hNinein) and SDA proteins CEP170 (ref. 13) and ODF2 (ref. 20; Fig. 1c–e). In the top and side views, the diameter of the ring formed by CCDC120 (491.0±42.5 nm diameter; Fig. 1c–f) was slightly smaller than that of hNinein (527±31.7 nm diameter; Fig. 1c,f) or CEP170 (550.3±15.8 nm diameter; Fig. 1d,f), but was much larger than that of ODF2 (374.5±28.6 nm; Fig. 1e,f). Similar results were obtained after overexpressing green fluorescent protein (GFP)-tagged CCDC120 (Supplementary Fig. 1j). We also co-immunostained CCDC120 with TCHP^23^ and CEP164 (ref. 30), markers of the subdistal parts of centrioles and DAs, respectively, and found that the diameter of the ring formed by CCDC120 was also larger than those formed by TCHP and CEP164 (Fig. 1g,h). Finally, immuno-EM results confirmed that CCDC120 is an SDA component (Fig. 1i). Taken together, these results show that CCDC120 is localized to the SDAs of mother centrioles and proximal ends of both centrioles in interphase cells (Fig. 1j).

### CCDC120 is recruited to SDAs by ODF2

**Figure 2:**
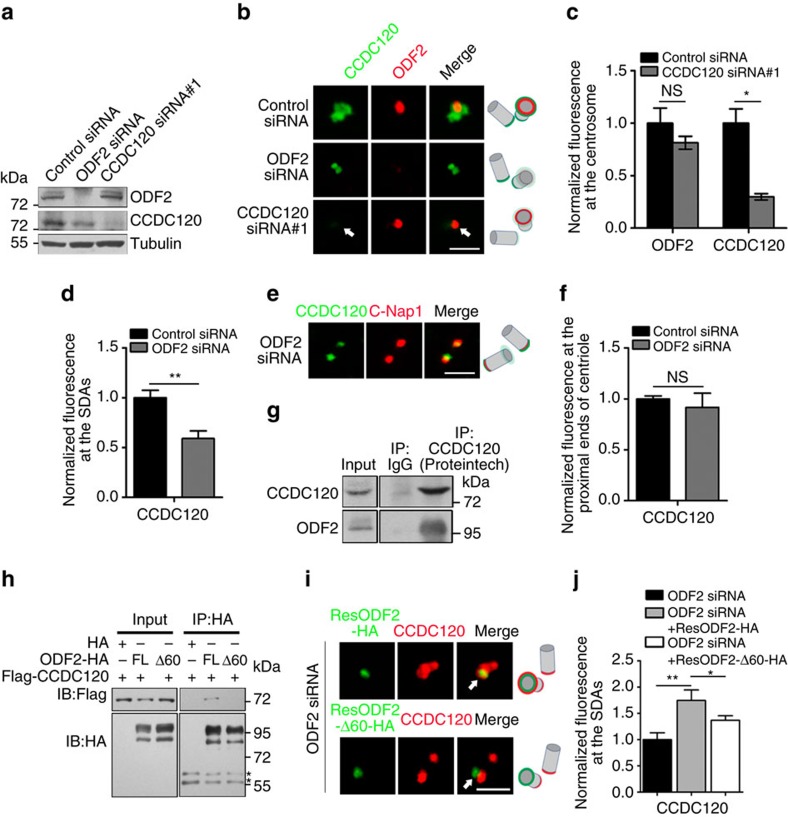
ODF2 recruits CCDC120 to SDAs. (**a**) Immunoblots showing depletion of ODF2 or CCDC120 by siRNA in U2OS cells. Tubulin was used as a loading control. (**b**) Immunostaining of CCDC120 (green) and ODF2 (red) in control-, ODF2- or CCDC120-siRNA-transfected U2OS cells. The arrows show centrosome localization. Scale bar, 1 μm. (**c**) Quantification of the fluorescence intensity of ODF2 and CCDC120 at the centrosomes from **b** (*n*>100 cells from three individual experiments). (**d**) Quantification of the fluorescence intensity of CCDC120 at the SDAs from **b** (*n*>100 cells from three individual experiments). (**e**) Immunostaining of CCDC120 (green) and C-Nap1 (red) in control- or ODF2-siRNA-transfected U2OS cells. Scale bar, 1 μm. (**f**) Quantification of the fluorescence intensity of CCDC120 at the proximal ends of the centrioles from **e** (*n*>100 cells from three individual experiments). (**g**) ODF2 and CCDC120 co-immunoprecipitated (IP) with anti-CCDC120 antibody (Proteintech) in lysates of HeLa cells. (**h**) Lysates of HEK293T cells co-overexpressing CCDC120-Flag and ODF2-HA full-length (FL) or those of the 1–60 aa deletion mutant (Δ60) were subjected to immunoprecipitation (IP) and immunoblotted (IB) with anti-HA and anti-Flag antibodies. The asterisks mark IgG bands. (**i**) Immunostaining of CCDC120 (red) and siRNA-resistant ODF2-HA (ResODF2-HA, green) or ResODF2-Δ60-HA (green) in ODF2-depleted U2OS cells. The arrows indicate SDA localization. Scale bar, 1 μm. (**j**) Quantification of the fluorescence intensity of CCDC120 at the SDAs from **i** (*n*>100 cells from three individual experiments). Data in **c**,**d**,**f** and **j** are the mean±s.e.m. Statistical significance was determined by a two-sided Student's *t*-test. *P<0.05, **P<0.01; NS, not significant. Unprocessed original scans of immunoblots are shown in Supplementary Fig. 7.

ODF2 is essential for SDA and DA assembly^20^. To investigate whether ODF2 is also required for localization of CCDC120 to SDAs, we examined the functional relationship between ODF2 and CCDC120. Depletion of ODF2 in U2OS cells by siRNA moderately reduced the protein level of CCDC120, and significantly inhibited localization of CCDC120 at SDAs (Fig. 2a–d), but not its localization at the proximal ends of centrioles (Fig. 2e,f). Depletion of CCDC120 by siRNA altered neither the protein level of ODF2 nor its centrosome localization (Fig. 2a–c). Furthermore, endogenous CCDC120 immunoprecipitated with ODF2 in HeLa cells (Fig. 2g and Supplementary Fig. 2a). These results suggest that ODF2 interacts with and recruits CCDC120 to SDAs.

To identify the region of ODF2 that mediates interaction with CCDC120, we constructed a series of ODF2 truncation mutants. Immunoprecipitation assays showed that 1–100 aa of ODF2 is required for its binding to CCDC120 (Supplementary Fig. 2b,c). Considering that 1–59 aa of ODF2 is important for SDA assembly in mouse cells^21^ as well as the high orthology between human and mouse ODF2, we generated a 1–60 aa deletion mutant of human ODF2 (Supplementary Fig. 2d). In comparison with full-length ODF2, the mutant showed significantly weakened interaction with CCDC120 (Fig. 2h). Moreover, overexpressing full-length, but not the 1–60 deletion mutant of siRNA-resistant ODF2, rescued SDA localization of CCDC120 in ODF2-depleted cells (Fig. 2i,j and Supplementary Fig. 2e). However, the interaction between ODF2 and CCDC120 was not detected by yeast two-hybrid assays (Supplementary Fig. 2f), suggesting that their interaction is indirect.

As TCHP localizes to the subdistal parts of both centrioles and mediates the interaction between ODF2 and hNinein^23^, we determined whether TCHP mediates the interaction between ODF2 and CCDC120. Depletion of TCHP did not alter both the total protein level and centrosome localization of CCDC120, and vice versa (Supplementary Fig. 2g–i), suggesting that TCHP is not required for SDA localization of CCDC120.

### CCDC120 recruits hNinein and CEP170 to centrosomes

**Figure 3:**
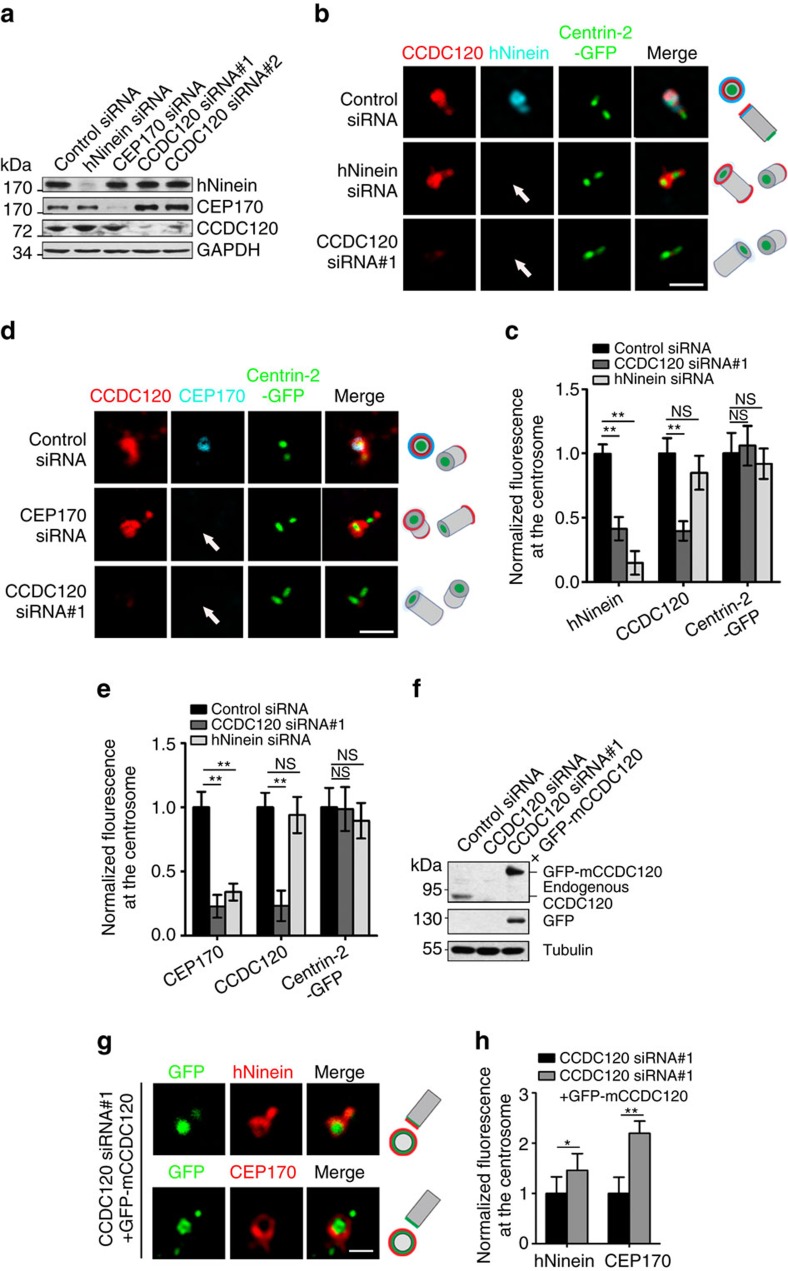
CCDC120 recruits CEP170 and hNinein to SDAs. (**a**) Immunoblots showing depletion of hNinein, CEP170 or CCDC120 by siRNA in U2OS cells. GAPDH was used as a loading control. (**b**) Immunostaining of CCDC120 (red) and hNinein (blue) in control-, hNinein- and CCDC120-siRNA-transfected Centrin-2-GFP (green)-overexpressed U2OS cells. The arrows indicate centrosome localization. Scale bar, 1 μm. (**c**) Quantification of the fluorescence intensity of hNinein, CCDC120 and Centrin-2-GFP at centrosomes from **b** (*n*>100 cells from three individual experiments). (**d**) Immunostaining of CCDC120 (red) and CEP170 (blue) in control-, CEP170- or CCDC120-siRNA-transfected Centrin-2-GFP (green)-overexpressed U2OS cells. The arrow indicates centrosome localization. Scale bar, 1 μm. (**e**) Quantification of the fluorescence intensity of CEP170, CCDC120 and Centrin-2-GFP at centrosomes from **d** (*n*>100 cells from three individual experiments). (**f**) Immunoblots show the siRNA-induced decrease of CCDC120, and rescued by GFP-tagged mouse CCDC120 (GFP-mCCDC120). Tubulin was used as a loading control. (**g**) Immunostaining of hNinein (red, upper) or CEP170 (red, lower) in CCDC120-depleted U2OS cells after transfection with GFP-mCCDC120. Scale bar, 500 nm. (**h**) Quantification of the fluorescence intensity of hNinein and CEP170 at centrosomes from **g** (*n*>100 cells from three individual experiments). Data in **c**,**e** and **h** are the mean±s.e.m. Statistical significance was determined by a two-sided Student's *t*-test. *P<0.05, **P<0.01; NS, not significant. Unprocessed original scans of immunoblots are shown in Supplementary Fig. 7.

To explore the function of CCDC120 in SDA assembly, we first depleted CCDC120 using siRNA and examined the centrosome localization of other SDA components. Loss of CCDC120 disrupted recruitment of hNinein and CEP170 to the centrosomes as indicated by Centrin-2, but not affecting their total protein levels (Fig. 3a–e). Overexpression of mouse CCDC120 in CCDC120-depleted U2OS cells rescued the SDA localization of CEP170 and hNinein (Fig. 3f–h). In contrast, depletion of either hNinein or CEP170 by siRNA did not affect both the centrosome localization and total protein level of CCDC120 (Fig. 3a–e). These results suggest that CCDC120 is necessary for recruitment of hNinein and CEP170 to SDAs. Moreover, depletion of CCDC120 affects neither the centrosome localization of DA protein CEP164 (Supplementary Fig. 3a–c) nor ciliogenesis in hTERT RPE-1 cells after serum starvation (Supplementary Fig. 3d–f), suggesting that CCDC120 is specifically involved in assembly of SDAs but not in assembly of DAs.

### hNinein and CEP170 bind to different domains of CCDC120

**Figure 4:**
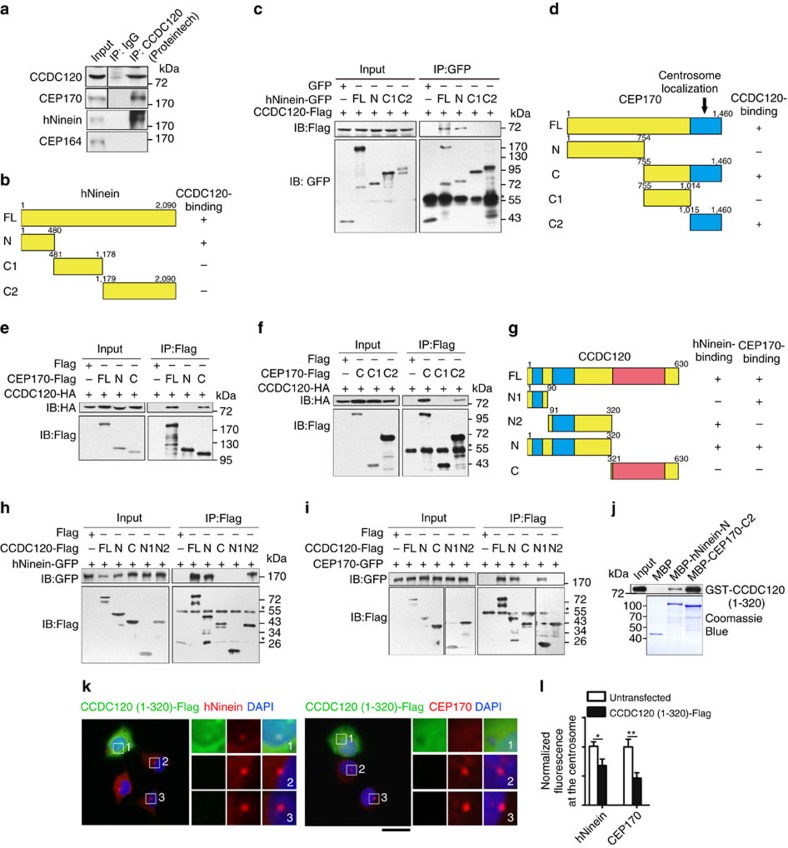
CCDC120 interacts with hNinein and CEP170 through two different coiled-coil domains. (**a**) Immunoprecipitation (IP) of CCDC120 with hNinein, CEP170 and CEP164 by anti-CCDC120 antibody (Proteintech) in lysates of HeLa cells. (**b**) Schematic of full-length (FL) hNinein and its truncates (N, C1 and C2). CCDC120-binding ability: +, positive; −, negative. (**c**) Lysates of HEK293T cells co-overexpressing CCDC120-Flag and hNinein-GFP truncates were subjected to IP and immunoblotted (IB) with anti-GFP and anti-Flag antibodies. The asterisk marks IgG band. (**d**) Schematic of FL CEP170 and its truncates (N, C, C1 and C2). The centrosome localization region is indicated in blue. CCDC120-binding ability: +, positive; −, negative. (**e**,**f**) Lysates of HEK293T cells co-overexpressing CCDC120-HA and the indicated CEP170-Flag truncates were subjected to IP and IB with anti-HA and anti-Flag antibodies. The asterisk marks IgG band. (**g**) Schematic of FL CCDC120 and its truncates (N1, N2, N and C). Coiled-coil domains, blue; proline-rich domains, red. CEP170- and/or hNinein-binding activity: +, positive; −, negative. (**h**,**i**) Lysates of HEK293T cells co-overexpressing hNinein-GFP (**h**) or CEP170-GFP (**i**) with the indicated CCDC120-Flag truncates were subjected to IP and IB with anti-GFP and anti-Flag antibodies. The asterisks mark IgG band. (**j**) *In vitro* binding assay. Purified GST-tagged CCDC120 (1–320 aa) was incubated with amylose resin beads coated with MBP, MBP-hNinein-N or MBP-CEP170-C2, and immunoblotted with anti-GST antibody. (**k**) Immunostaining of Flag (green), hNinein (red; left) or CEP170 (red; right) in U2OS cells overexpressing Flag-tagged CCDC120 (1–320 aa). DNA was stained with 4,6-diamidino-2-phenylindole (DAPI; blue). The centrosome localizations are magnified. Scale bars, 5 μm. (**l**) Quantification of the fluorescence intensity of hNinein and CEP170 at centrosomes from **k** (*n*>100 cells from three individual experiments). Data are the mean±s.e.m. Statistical significance was determined by a two-sided Student's *t*-test. *P<0.05, **P<0.01; NS, not significant. Unprocessed original scans of immunoblots are shown in Supplementary Fig. 7.

To further examine the relationships among CCDC120, CEP170 and hNinein, we coexpressed CCDC120-mCherry with hNinein-GFP or CEP170-GFP in U2OS cells. CCDC120-mCherry showed good co-localization with hNinein-GFP as aggregates throughout the cytoplasm (Supplementary Fig. 4a), and co-localized with microtubule-binding filaments formed by CEP170-GFP (Supplementary Fig. 4b). Next, immunoprecipitation assays showed that endogenous hNinein and CEP170, but not CEP164, bound to CCDC120 (Fig. 4a and Supplementary Fig. 4c). Furthermore, we mapped the domains of hNinein and CEP170 mediating the interactions with CCDC120, revealing that the N terminus (1–480 aa) of hNinein and C terminus (1,015–1,460 aa) of CEP170 were required for their binding to CCDC120 (Fig. 4b–f). Reciprocal immunoprecipitation was performed to identify the regions of CCDC120 responsible for associating with hNinein and CEP170. The N terminus of CCDC120 (1–320 aa) was able to bind hNinein and CEP170 (Fig. 4g–i). We further confirmed that both purified 1–480 aa of hNinein and 1,015–1,460 aa of CEP170 bound to 1–320 aa of CCDC120 *in vitro* (Fig. 4j). Overexpression of 1–320 aa of CCDC120 resulted in a marked decrease in the centrosome localization of endogenous hNinein and CEP170, but not that of CEP164, in U2OS cells (Fig. 4k,l and Supplementary Fig. 4d,e). These results suggest that the N terminus of CCDC120 is required for SDA localization of hNinein and CEP170.

To map the hNinein- and CEP170-binding regions of CCDC120 in more detail, we performed yeast two-hybrid assays using two coiled-coil domains of CCDC120 as bait (Fig. 4g). The first coiled-coil domain-containing region (1–90 aa) of CCDC120 bound to the C terminus of CEP170 (1,015–1,460 aa), while its second coiled-coil domain-containing region (91–320 aa) interacted with the N terminus of hNinein (1–480 aa, Supplementary Fig. 4f). Immunoprecipitation and glutathione *S*-transferase (GST) pull-down assays further confirmed that the two coiled-coil domain-containing regions of CCDC120 at 1–90 aa and 91–320 aa bound to CEP170 and hNinein, respectively (Fig. 4h,i and Supplementary Fig. 4g,h).

Collectively, these results suggest that CCDC120 interacts with hNinein and CEP170, most likely in a direct manner, through different coiled-coil domains at its N terminus.

### CCDC68 is a CEP170-interacting protein localized to SDAs

**Figure 5:**
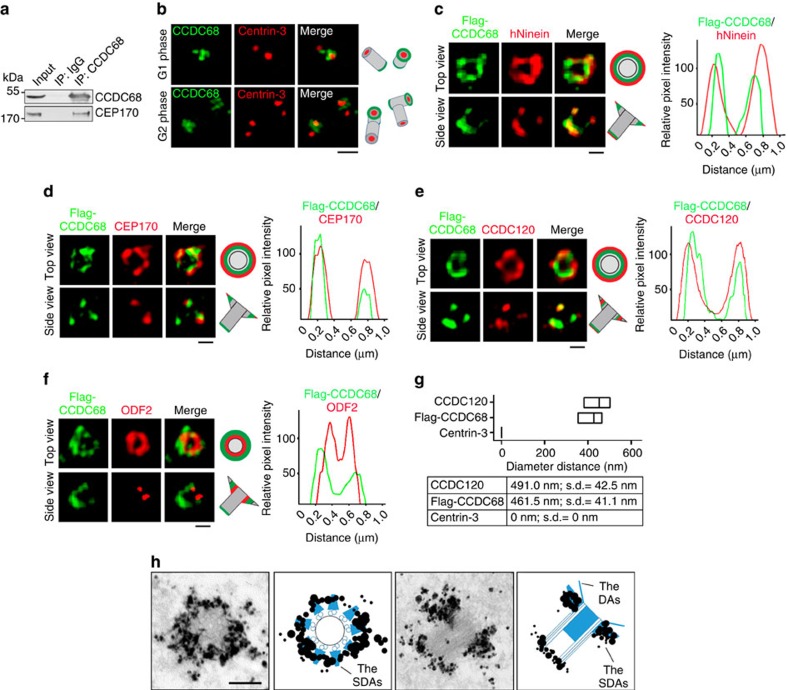
CCDC68 interacts with CEP170 and is localized at the centrosomes. (**a**) Immunoprecipitation (IP) of CCDC68 with CEP170 in lysates of HeLa cells. (**b**) Immunofluorescence of CCDC68 (green) and Centrin-3 (red) in U2OS cells. Scale bar, 500 nm. (**c**–**f**) 3D-SIM images of Flag-CCDC68-overexpressing U2OS cells double-immunostained with antibodies against Flag (green) and hNinein (**c**; red), CEP170 (**d**, red), CCDC120 (**e**, red) or ODF2 (**f**, red). Scale bars, 500 nm. The intensity plots of the rings are, respectively, shown at right. (**g**) Average diameter of the ring-like structure formed by the listed proteins. The low-high bars (horizontal) show the range of the diameter and the vertical lines indicate the mean. From the bottom to the top: *n*=10; 36; and 28. (**h**) Immuno-EM images. U2OS cells were labelled with anti-CCDC68 antibody followed by nanogold-coupled secondary antibody. Schematics of immuno-EM images are shown. Scale bar, 200 nm. Supplementary Table 1 lists the CEP170-interacting proteins.

To identify additional components participating in SDA assembly, we screened CEP170-interacting proteins by mass spectrometry analysis and chose to focus on CCDC68 (Supplementary Table 1). A specific band at ∼45 kDa was detected by anti-CCDC68 antibody in U2OS cells; this band was diminished after CCDC68 depletion (Supplementary Fig. 5a). The interaction between CCDC68 and CEP170 was confirmed by immunoprecipitation with the anti-CCDC68 antibody (Fig. 5a). Next, we co-immunostained CCDC68 with Centrin-3 in U2OS cells, revealing asymmetric localization of CCDC68 on the two centrioles in G1 phase cells (Fig. 5b, top), similar to that of CCDC120 (Fig. 1a) and other SDA proteins^11^,^13^. Also, the asymmetric localization of CCDC68 became symmetrical, followed by maturation of old daughter centrioles in G2 phase cells (Fig. 5b, bottom).

To investigate the detailed localization of CCDC68 at the centrosomes, we co-immunostained CCDC68 with SDA proteins hNinein, CEP170, ODF2 and CCDC120, and observed them under 3D-SIM. Both endogenous and Flag-tagged CCDC68 formed a ring-like structure at the distal part of the mother centriole similar to those formed by CCDC120 and hNinein (Fig. 5c–f and Supplementary Fig. 5b). The CCDC68 ring was co-localized with the detected SDA proteins in the top and side views; moreover, it was localized to the proximal parts of both centrioles (Fig. 5b–f). The diameter of the ring formed by CCDC68 (461.5±41.1 nm diameter) was slightly smaller than that of hNinein, CEP170 or CCDC120, but larger than that of ODF2 (Fig. 5c–g). Furthermore, we confirmed that CCDC68 is an SDA component by immuno-EM (Fig. 5h).

### CCDC68 and CCDC120 independently recruit CEP170

**Figure 6:**
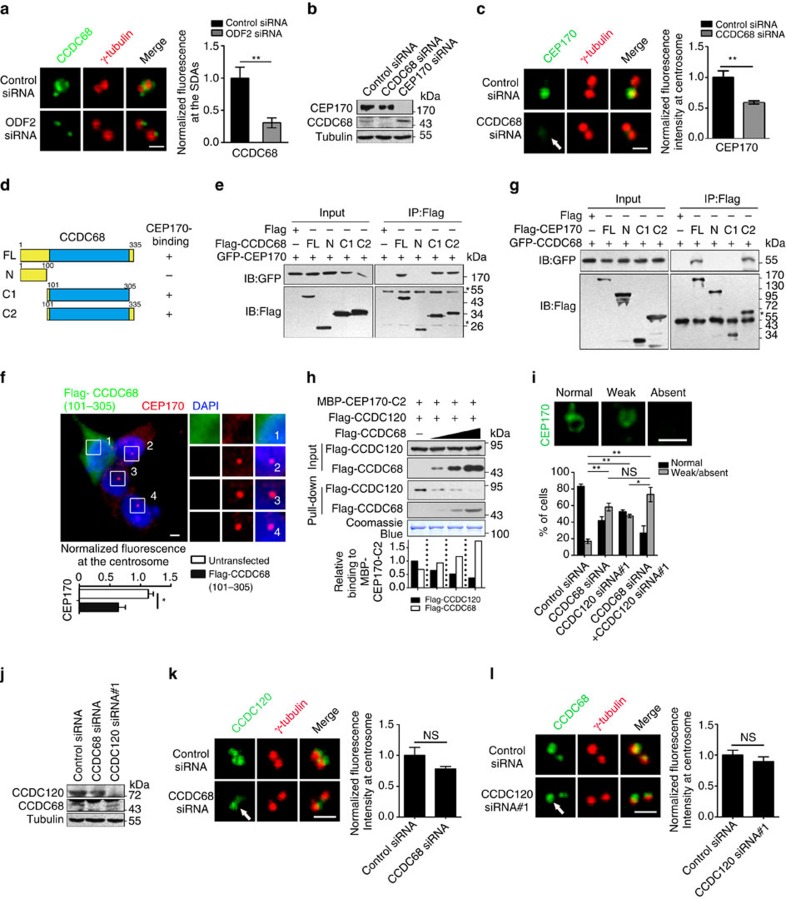
CCDC68 and CCDC120 competitively interact with CEP170. (**a**) Immunostaining of CCDC68 (green) and γ-tubulin (red) in control- or ODF2-siRNA-transfected U2OS cells. Quantification of CCDC68 at the SDAs are shown at right. (**b**) Immunoblots show depletion of CCDC68 and CEP170 by siRNA in U2OS cells. (**c**) Immunostaining of CEP170 (green) and γ-tubulin (red) in control- or CCDC68-siRNA-transfected U2OS cells. Arrow, centrosome localization. Quantification of CEP170 at centrosomes are shown at right. (**d**) Schematic of CCDC68. FL, full-length; coiled-coil domains, blue; +, positive; −, negative. (**e**) Lysates of HEK293T cells co-overexpressing GFP-CEP170 and Flag-CCDC68 truncates were subjected to immunoprecipitation (IP) and immunoblotted (IB) with the indicated antibodies. Asterisks, IgG bands. (**f**) Immunostaining of U2OS cells overexpressing Flag-CCDC68 (101–305 aa) with anti-Flag (green) and anti-CEP170 (red) antibodies. DNA, 4,6-diamidino-2-phenylindole (DAPI; blue). Centrosomes are magnified. Quantification of CEP170 at centrosomes is shown below. (**g**) Lysates of HEK293T cells co-overexpressing GFP-CEP170 and Flag-CCDC68 truncates were subjected to IP and IB with anti-GFP and anti-Flag antibodies. Asterisk, IgG. (**h**) Lysates of HEK293T cells overexpressing Flag-CCDC120 or Flag-CCDC68 were subjected to pull-down assays with MBP-CEP170-C2 (1,015–1,460 aa). Quantification of the Flag-CCDC68 and Flag-CCDC120 band intensity (normalized to MBP-CEP170-C2) in three independent experiments are shown on the graph. (**i**) Quantification of the fluorescence intensity of CEP170 at centrosomes in the indicated siRNA-transfected U2OS cells. (**j**) Immunoblots show depletion of CCDC68 and CCDC120 by siRNA in U2OS cells. (**k**) Immunostaining of CCDC120 (green) and γ-tubulin (red) in control- or CCDC68-siRNA-transfected U2OS cells. Arrow, centrosome localization. Quantification of CCDC120 at centrosomes are shown at right. (**l**) Immunostaining of CCDC68 (green) and γ-tubulin (red) in control- or CCDC120-siRNA-transfected U2OS cells. Arrow, centrosome localization. Quantification of CCDC68 at centrosomes are shown at right. For **a**,**c**,**f**,**i**,**k** and **l** scale bars, 1 μm. Data in **a**,**c**,**f**,**i**,**k** and **l** are the mean±s.e.m. *n*>100 cells from three individual experiments, Statistical significance was determined by a two-sided Student's *t*-test. *P<0.05, **P<0.01; NS, not significant. Unprocessed original scans of immunoblots are shown in Supplementary Fig. 7.

Next, we performed experiments aimed at clarifying the function of CCDC68 in SDAs assembly. First, loss of ODF2 strikingly weakened the SDAs localization of CCDC68 in U2OS cells (Fig. 6a and Supplementary Fig. 5c), although the interaction between ODF2 and CCDC68 was barely detected (Supplementary Fig. 5d), suggesting that ODF2 does not directly recruit CCDC68 to SDAs. Because CCDC68 interacts with CEP170 and the diameter of the CCDC68 ring at SDAs is slightly smaller than that of CEP170 (Fig. 5a,d), we assessed whether CCDC68 also recruits CEP170 to SDAs. Depletion of CCDC68 in U2OS cells by siRNA resulted in ∼40% loss of the CEP170 signal at the centrosomes without changing its protein level (Fig. 6b,c), whereas loss of CEP170 altered neither the localization of CCDC68 at the centrosome nor the protein level of CCDC68 (Fig. 6b and Supplementary Fig. 5e), suggesting that CCDC68 is involved in recruiting CEP170 to SDAs. In addition, cilia formation was not affected after depletion of CCDC68 in hTERT RPE-1 cells (Supplementary Fig. 5f–h).

To determine which domains mediate the interaction between CCDC68 and CEP170, we constructed several truncation mutants of CCDC68. Immunoprecipitation showed that the truncates of CCDC68 containing a long coiled-coil domain in the middle region (101–305 aa) interacted with CEP170 (Fig. 6d,e). When 101–305 aa of CCDC68 was overexpressed in U2OS cells, the centrosome localization of endogenous CEP170 was remarkably diminished (Fig. 6f), similar to the phenotype observed in the cells overexpressing the N terminus of CCDC120 (1–320 aa, Fig. 4k). We also mapped the CEP170-interacting domain of CCDC68 in detail using yeast two-hybrid assay, revealing that 101–200 aa of CCDC68 mainly bound to CEP170 (Supplementary Fig. 6a,b).

An unstable isoform of CCDC68 (CCDC68Δ69–114) that lacks tumour-suppressive ability has been reported in 31% of pancreatic ductal adenocarcinoma patients^37^. In comparison with full-length CCDC68, CCDC68Δ69–114 showed similar SDA localization and CEP170-binding ability (Supplementary Fig. 6c–e), suggesting that the tumour-suppressive function of CCDC68 may not rely on its functions at SDAs.

Next, we investigated the domains of CEP170 involved in its association with CCDC68. Unexpectedly, the C terminus of CEP170 (1,015–1,460 aa), which was shown to mediated the centrosome localization of CEP170 and its interaction with CCDC120 (Fig. 4f), also co-immunoprecipitated with CCDC68 (Fig. 6g and Supplementary Fig. 6f). To determine whether CCDC120 and CCDC68 recruit CEP170 to SDAs in a competitive manner, we incubated the purified C terminus of CEP170 sequentially with lysates from HEK293T cells individually overexpressing a constant amount of CCDC120 and an increasing amount of CCDC68. The results showed that increased binding of CCDC68 to the C terminus of CEP170 was associated with reduced interaction between CCDC120 and the C terminus of CEP170 (Fig. 6h). In contrast, increasing CCDC120 level barely displaced CCDC68 from the C terminus of CEP170 (Supplementary Fig. 6g). These data suggest that CCDC68 and CCDC120 bind to the same region, or to overlapping regions, in the C terminus of CEP170 in a competitive manner.

The competitive binding of CCDC68 and CCDC120 to CEP170 suggests that they may function independently in SDA assembly. Indeed, compared with depleting CCDC120 alone, double-knockdown CCDC68 and CCDC120 further impaired centrosome localization of CEP170 in U2OS cells (Fig. 6i and Supplementary Fig. 6h). However, the interaction between CCDC68 and CCDC120 was barely detected (Supplementary Fig. 6i), and localization of CCDC68 at SDAs was not dependent on the presence of CCDC120 and vice versa (Fig. 6j–l). Together, these results reveal that CCDC120 and CCDC68 function independently in recruiting CEP170 and SDA localization.

### CCDC120 and CCDC68 are required for microtubule anchoring

**Figure 7:**
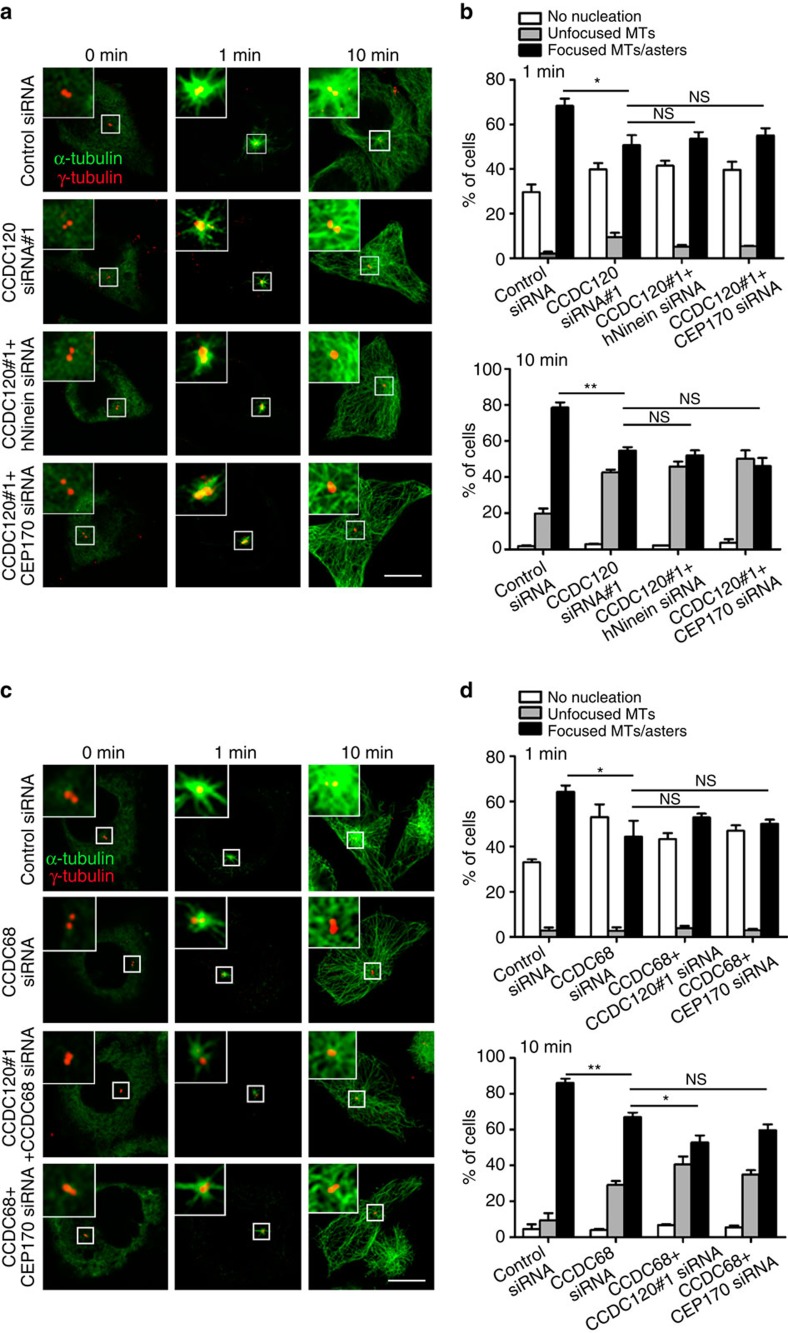
Depletion of CCDC120 and CCDC68 impairs microtubule anchoring in interphase cells. (**a**) Immunostaining of α-tubulin (green) and γ-tubulin (red) in control-, CCDC120-, CCDC120/hNinein- or CCDC120/CEP170-siRNA-transfected HeLa cells after microtubule regrowth for the indicated time periods. Scale bar, 5 μm. (**b**) Quantification of cells with different microtubule regrowth statuses from **a** (*n*>100 cells from three individual experiments). (**c**) Immunostaining of α-tubulin (green) and γ-tubulin (red) in control-, CCDC68-, CCDC68/CCDC120- or CCDC68/CEP170-siRNA-transfected HeLa cells after microtubule regrowth for the indicated time periods. Scale bar, 5 μm. (**d**) Quantification of cells with different microtubule regrowth statuses from **c** (*n*>100 cells from three individual experiments). Data in **b** and **d** are the mean±s.e.m. Statistical significance was determined by a two-sided Student's *t*-test. *P<0.05, **P<0.01; NS, not significant.

On the basis of the localization of CCDC120 and CCDC68 at SDAs and their relationships among hNinein, CEP170 and ODF2, which are essential for microtubule anchoring to SDAs^18^,^20^,^21^, we determined the functions of CCDC120 and CCDC68 in microtubule organization at the centrosome. First, flow cytometry analysis showed that depletion of neither CCDC120 nor CCDC68 significantly affected the cell cycle (Supplementary Fig. 6j). Next, microtubules were depolymerized by cold treatment and allowed to recover after the addition of warm medium to the cells. After 1 min of recovery, strong microtubule asters were observed in the control cells, whereas fewer and weaker asters were found in the CCDC120- and CCDC68-depleted cells (1 min, Fig 7a–d). Ten minutes after regrowth, cytoplasmic microtubules had grown well in the cells in each group. However, in the CCDC120- and CCDC68-knockdown cells, the number of cells with microtubules anchored to the centrosome was significantly decreased in comparison with that of the control cells (10 min, Fig. 7a–d). Microtubule-anchoring defects of greater severity than those observed in either single depletion group were observed in the CCDC120/CCDC68 double-knockdown cells (Fig. 7c,d). Thus, CCDC120 and CCDC68 function independently and both are required for centrosome microtubule anchoring in interphase cells.

Because CCDC120 interacts with hNinein and CEP170 (Fig. 4), while CCDC68 associates with CEP170 (Fig. 6b–l), we performed microtubule regrowth assays in cells subjected to double knockdown of CCDC120/hNinein, CCDC120/CEP170 or CCDC68/CEP170. In these double-knockdown cells, the percentage of cells showing microtubule anchoring to the centrosome did not decrease significantly after 10 min of recovery, in comparison with the percentages of CCDC120 and CCDC68 single-knockdown cells showing such anchoring (Fig. 7a–d). Together, these results suggest that CCDC120 promotes centrosome microtubule anchoring in interphase cells mediated by hNinein and CEP170, while CCDC68 functions mainly requiring CEP170.

## Discussion

**Figure 8:**
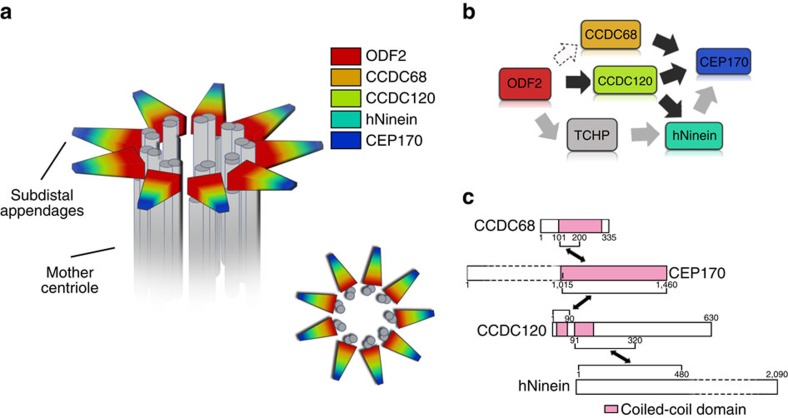
Schematic model of the roles of CCDC120 and CCDC68 in hierarchical SDA assembly in interphase cells. (**a**) Schematic model of SDAs. SDA components ODF2, CCDC68, CCDC120, hNinein and CEP170 formed a ring-like structure at SDAs of the mother centrioles in interphase cells. (**b**) Schematic model of SDA assembly. ODF2 acts upstream to initiate SDA assembly^20^,^21^. TCHP mediates the interaction between ODF2 and hNinein^23^. CEP170 can be recruited by hNinein^19^. CCDC120 is recruited to SDAs by ODF2 and recruits both CEP170 and hNinein to SDAs through two distinct domains. CCDC68 competes with CCDC120 in recruiting CEP170. (**c**) Schematic model of the interactions of CCDC68, CEP170, CCDC120 and hNinein, and their binding domains.

In this study we identified two new SDA components, CCDC120 and CCDC68, which cooperate with known SDA components ODF2, hNinein and CEP170, and are required for hierarchical SDA assembly and microtubule anchoring in interphase cells (Fig. 8).

Like other SDA proteins such as ODF2 (ref. 21), CEP170 (ref. 13) and hNinein^11^, CCDC120 and CCDC68 form a ring structure during the G1 phase; two rings appear during the G2 phase, as the old daughter centriole matures. Using 3D-SIM, we showed that the rings formed by CCDC120 and CCDC68 at SDAs are a similar diameter, and much larger than that of ODF2, but slightly smaller than those of hNinein and CEP170. These results suggest that the protein rings formed by SDA components ODF2, CCDC68, CCDC120, hNinein and CEP170 overlapped to form a ring-like structure at the SDAs of mother centrioles in interphase cells (Fig. 8a).

ODF2 is essential for organization of DAs and SDAs via the actions of two distinct fragments. Amino acids 188–806 of mouse ODF2 are necessary for DA formation, whereas SDA formation requires 1–59 aa and 188–806 aa regions^20^,^21^. Interestingly, we found that 1–60 aa of human ODF2, which is highly orthologous to 1–60 aa of mouse ODF2, recruits CCDC120 to SDAs without affecting its localization at the proximal ends of centrioles (Fig. 2). However, we did not detect a protein–protein interaction between CCDC68 and ODF2, suggesting that other proteins may mediate the interaction between these two components.

TCHP acts as a bridge between ODF2 and hNinein at SDAs^23^. However, we found no detectable effect of TCHP on centrosome localization of CCDC120 or vice versa, suggesting that hNinein may be simultaneously recruited by CCDC120 and TCHP at different domains. In mouse cells, Ninein targets the centrosome through its C terminus, whereas at least four centrosome target sites of hNinein have been found in human cells^38^,^39^,^40^,^41^. We showed that CCDC120 interacts with 1–480 aa of hNinein, one of hNinein centrosome target sites^38^. Therefore, it is possible that hNinein may be recruited by TCHP through its other centrosome target sites.

CEP170 is recruited by hNinein in human cells, as the C terminus of CEP170 directly interacts with the middle region and C terminus of hNinein^19^. Here our results suggest that the N terminus of CCDC120 interacts with hNinein and CEP170, most likely directly, through two distinct coiled-coil domains; the first interacts with the C terminus of CEP170 (1,015–1,460 aa), whereas the second interacts mainly with the N terminus of hNinein (1–480 aa). These results are consistent with a previous report that the N terminus of hNinein mediates its centrosome localization^41^, as well as with a report that the C terminus of CEP170 is essential for its targeting to the centrosome^13^. Interestingly, we found that compared with CCDC120, CCDC68 shows more dominant in binding the C terminus of CEP170, as CCDC68 efficiently outcompeted CCDC120 for the C terminus of CEP170 in the binding assays. In addition, we did not detect strong interaction or direct recruitment relationships between them, further demonstrating their independent functions in SDA assembly. Future investigations should assess whether CCDC120 and CCDC68 sequentially recruit CEP170 during maturation of SDAs in mother centrioles. Thus, our results suggest that SDA assembly might be a hierarchical process with downstream branching, rather than a simple linear process (Fig. 8b,c).

The relationship between SDA components and cilia formation has been the subject of several studies. ODF2 is required for primary cilia formation in fibroblasts^33^ and proper basal bodies' organization at the cell cortex in multiple ciliated cells^42^. Loss of SDA protein CC2D2A causes deficient cilia formation and SDA localization of ODF2 (ref. 15). Interestingly, a recent study reported that the loss of SDA components such as ODF2, CEP170 and Ninein change neither the length of primary cilia nor the percentage of ciliated cells, but affects the spatial configuration of cilia^34^. In this study, our data also showed that depletion of CCDC120 and CCDC68 changes neither the percentage of ciliated cells nor the ciliary length. Future investigations should assess the effects of depletion of CCDC120 or CCDC68 on ciliary position and signal sensing.

Similar to CEP170 and Ninein^11^,^18^,^19^, CCDC120 and CCDC68 are required for microtubule anchoring at centrosomes. Our preliminary data did not show any microtubule-binding activity of CCDC120 or CCDC68; therefore, CCDC120 may indirectly regulate microtubule organization through hNinein and CEP170, while the functions of CCDC68 in microtubule anchoring are mainly mediated by CEP170. Furthermore, loss of CCDC120 or/and CCDC68 also affected microtubule nucleation at the early stage of microtubule regrowth (Fig. 7, 1 min), indicating that CCDC120 and CCDC68 may also be involved in microtubule nucleation processing. Consistently, depletion of microtubule-anchoring proteins such as EB1, Kif3a and p150^Glued^ delays microtubule nucleation^22^,^24^,^25^, suggesting a close relationship between microtubule nucleation and anchoring.

## Methods

### Plasmid construction

Mouse CCDC120 (NM_207202.2), human CCDC120 (NM_001271836.1), human CCDC68 (NM_025214.2) and all CCDC120 and CCDC68 truncations were amplified by PCR and cloned into pEGFP-N3 (Clontech), pcDNA3.1-HA and p3 × Flag-CMV-14 (Sigma-Aldrich). Human Ninein and CEP170 were PCR-amplified from plasmids provided by Erich A. Nigg (University of Basel, Basel, Switzerland) and cloned into pEGFP-N3 (Clontech). Human ODF2 was PCR-amplified and cloned into pcDNA3.1-HA. CEP170 was PCR-amplified and cloned into pEGFP-N3 (Clontech) and p3 × Flag-CMV-14 (Sigma-Aldrich). Human Centrin-2 (NM_004344.1) was amplified by PCR and cloned into pEGFP-N3 (Clontech).

### Antibodies

All antibodies used in this study are listed in Supplementary Tables 2 and 3.

### Cell cultures and treatments

HeLa and HEK293T cells were purchased from American Type Culture Collection. U2OS and hTERT RPE-1 cells were provided by Xueliang Zhu at SIBS, Chinese Academy of Sciences^43^,^44^. HeLa, U2OS and HEK293T cells were cultured in DMEM (GIBCO) with 10% fetal bovine serum (GIBCO or CellMax). hTERT RPE-1 cells were cultured in DMEM/F12 (1:1, GIBCO) with 10% fetal bovine serum. All cells were cultured at 37 °C in an atmosphere containing 5% CO_2_. HEK293T cells were transfected by the standard calcium phosphate method, whereas the other types of cells were transfected with jetPEI (Polyplus transfection) or Lipofectamine 2000 (Invitrogen) according to the manufacturer's instructions.

For mitosis synchronization, HeLa cells were treated with 100 ng ml^−1^ nocodazole for 24 h. For double-thymidine blocking, HeLa cells were treated with 2.5 mM thymidine for 18–24 h, released for 12 h and blocked again for 18–24 h. To induce primary cilia, hTERT RPE-1 cells were cultured in DMEM/F12 without serum for 48 h.

### Gene silencing by siRNA

The siRNAs used in this study were obtained from Invitrogen and consisted of the following sequences: CCDC120 #1, 5′-GGGAGUGGCUAGUCAUGAUTT-3′; CCDC120 #2, 5′-CAGCUCUCCUACCUUCAAUTT-3′; hNinein, 5′-GGAAGAAUAUCGUGCACAATT-3′; CEP170, 5′-GAAGGAAUCCUCCAAGUCATT-3′; ODF2, 5′-GGUCACUGUAAAAUGAACCTT-3′; TCHP, 5′-GGCAGAAUGGAGCUCUAAATT-3′; CCDC68, 5′-CUGCGUGAGUCUUAUUUAUTT-3′; and negative control siRNA, 5′-UUCUCCGAACGUGUGUCACGU-3′. All siRNAs were transfected by Lipofectamine 2000 (Invitrogen) according to the manufacturer's instructions and analysed 48 h after transfection^45^. To generate siRNA-resistant ODF2 (resODF2), five silent mutations were introduced into the ODF2 sequence by siRNA (5′-GGTCACTGTAAAATGAACC-3′ was mutated to 5′-GGACATTGCAAGATGAATC-3′).

### Immunofluorescence and immuno-EM

Cells were fixed and permeabilized in methanol for 5–10 min at −20 °C, incubated with first antibodies in PBS with 4% BSA at 4 °C overnight, then stained by secondary antibodies and 1 μg ml^−1^ 4,6-diamidino-2-phenylindole^45^. The antibodies used in the immunofluorescence are shown in Supplementary Tables 2 and 3. The samples were observed at room temperature using a fluorescence microscope (TH4-200, Olympus) equipped with a 60 × 1.42 numerical aperture (NA) Apo Oil Objective lens (Olympus), a confocal microscope (LSM-710 NLO, Zeiss) equipped with a 100 × 1.4 NA Apo Oil Objective lens (C-Apochromat, Zeiss) or a confocal microscope (Leica TCS SP8) equipped with a 100 × 1.4 NA Objective lens. Images were acquired by DP controller software (Olympus) or ZEN 2009 software (Zeiss). Confocal image deconvolution was acquired by Huygens software (Scientific Volume Imaging, Netherlands). 3D-SIM was performed using an N-SIM System (Nikon) equipped with a 100 × 1.49 NA Apo Oil Objective lens (Nikon) at room temperature. All images were reconstructed to maximum projections using NIS-Elements AR software (Nikon). Image processing was performed in Photoshop (CC; Adobe).

For Immuno-EM^45^, U2OS cells were fixed with 4% paraformaldehyde (PFA) in PBS with 0.2% Triton X-100 for 10 min, and incubated with anti-CCDC120 (lab-generated, rabbit) or anti-CCDC68 (GeneTex, GTX106883) primary antibody in PB (phosphate buffer, 0.2M Na_2_HPO_4_, 0.2M NaH_2_PO_4_; pH 7.4) at 4 °C overnight, after which they were incubated with goat anti-rabbit IgG-nanogold secondary antibody (1:40, Nanoprobes). HQ Silver (Nanoprobes) was used to enhance the nanogold signal for ∼12 min. Samples were post-fixed in 0.5% osmium tetroxide in phosphate buffer for 15 min, dehydrated and embedded in Epon. Finally, immunogold-stained sample sections were stained with 4% aqueous uranyl acetate and 0.4% lead citrate, and imaged with a transmission electron microscope (FEI, Tecnai G2 20 Twin).

### Immunoprecipitation and pull-down assay

For exogenous immunoprecipitation, HEK293T cells were washed three times with cold PBS and lysed in immunoprecipitation lysis buffer (50 mM HEPES, 0.1% NP-40, 250 mM NaCl, 1 mM dithiothreitol, 5 mM EDTA, 10% glycerol and protease inhibitors; pH 7.5) on ice for 30 min after transfection for 36 h. Appropriate antibodies were incubated with protein G-Sepharose beads (Amersham Biosciences) for 2 h, followed by incubation with the supernatants of the lysates (centrifuged at 20,000*g* for 20 min at 4 °C) for 3 h. For endogenous immunoprecipitation, HeLa cells were washed with PBS and lysed in lysis buffer (50 mM HEPES, 150 mM NaCl, 1 mM EDTA, 0.5% Triton X-100 and protease inhibitors; pH 7.4) on ice for 30 min. Appropriate lab-generated or commercial (Proteintech, 22041-AP) rabbit polyclonal antibody against CCDC120 or commercial antibody against CCDC68 (GeneTex, GTX106883) were individually incubated with protein A-Sepharose beads (Amersham Biosciences) for 2 h, followed by incubation with the supernatants of the lysates (centrifuged at 20,000*g* for 15 min at 4 °C) for 2 h. After washing the beads with lysis buffer, they were collected and boiled at 100 °C for 5 min in SDS loading buffer. For λ-PPase treatment, cell lysates were treated with λ-PPase (NEB) for 30 min according to the manufacturer's instructions.

For pull-down assays, purified GST- or maltose binding protein (MBP)-tagged fusion proteins were incubated with glutathione-Sepharose 4B beads (GE Healthcare) or amylose resin beads (NEB) at 4 °C for 1–2 h, respectively. After washing the beads several times with PBS, they were incubated with the supernatants from the cell lysates or purified GST-tagged fusion proteins at 4 °C for at least 2 h. After washing the beads with lysis buffer, they were collected and boiled at 100 °C for 5 min in SDS loading buffer.

For sequential pull-down assays, amylose resin beads (NEB) were incubated with purified MBP-CEP170-C2 proteins at 4 °C for 1 h. After washing, the beads were incubated with the lysates of HEK293T cells overexpressing Flag-CCDC120 (or Flag-CCDC68) at 4 °C for 1 h, and sequentially incubated with the lysates of HEK293T cells overexpressing Flag-CCDC68 (or Flag-CCDC120) at 4 °C for 1 h after washing the beads. Finally, the beads were washed and boiled in SDS loading buffer.

For immunoblotting, samples were resolved by SDS–PAGE and transferred to a polyvinylidene difluoride membrane (Millipore). The membrane was incubated with primary and secondary antibodies (showed in Supplementary Tables 2 and 3).

### Yeast two-hybrid assay

CCDC120, CEP170, hNinein and ODF2 truncation mutants were cloned into bait vector pGBKT7 (Clonetech) or prey vector pGADT7 (Clonetech). Yeast two-hybrid experiments were performed following the instructions in the Matchmaker Two-Hybrid System Handbook. Briefly, the prey and bait vectors were transformed together into AH109 yeast strains, which were plated onto double (Trp- and Leu-) and quadruple (Trp-, Leu-, His- and Ade-) selective media for 2–4 days at 30 °C.

### Microtubule regrowth assay

HeLa cells were treated with cold medium on ice for 30 min to depolymerize microtubules. Microtubule regrowth was allowed in warm medium for several minutes. The cells were washed with warm PBS to remove the medium and treated with PBS containing 2% PFA, 0.05% Triton X-100 and 10 μM Taxol for 2 min at room temperature. After washing with PBS, the cells were fixed in 4% PFA at 37 °C for 15 min. Next, the cells were washed several times with PBS and stained with anti-α-tubulin and anti-γ-tubulin antibodies.

### Flow cytometry analysis and centrosome isolation

For flow cytometry, cells were trypsinized and fixed in cold 70% ethanol, which was added dropwise. Next, the fixed cells were stained with propidium iodide for 1 h and analysed using FACScalibur (BD Biosciences) and CellQuest software.

For centrosome isolation, HeLa cells were treated with 10 μg ml^−1^ nocodazole for 1 h. Next, the cells were lysed in lysis buffer (1 mM HEPES, 0.5% NP-40, 0.1% β-mercaptoethanol, 0.5 mM MgCl_2_ and protease inhibitors; pH 7.2) and centrifuged at 1,200*g* for 10 min at 4 °C, after which the supernatant was collected and mixed with 100 μl of 1 M HEPES and 20 μl of 1 μM DNase I and incubated for 30 min at 4 °C. The sample was added to a centrifuge tube containing 0.5 ml of 70% sucrose buffer, 0.3 ml of 50% sucrose buffer and 0.3 ml of 30% sucrose buffer, after which it was centrifuged at 40,000*g* for 1 h at 4 °C. The fractions were collected from the bottom of the 70% sucrose layer.

### Measurements and statistical analysis

The intensities of immunofluorescence and immunoblotting bands were measured using Scion Image and Fiji software. NIS-Elements AR software (Nikon) was used to calculate the diameters of the ring-like structures formed by centrosome proteins and to make plot profiles. For measuring the primary cilia length, primary cilia were labelled by antibody against Arl13b and imaged by using *z*-stack confocal microscopy. The thickness of *z*-slices and projection length of cilia on the *x*–*y* plane formed two right-angle sides of the right triangle, and the hypotenuse is the cilia length^46^. Statistical significance was determined by the two-sided Student's *t*-test. All experiments were performed three times.

### Data availability

The data that support the findings of this study are available from the corresponding authors on reasonable request. Full scans of immunoblots are available in Supplementary Fig. 7.

## Additional information

**How to cite this article:** Huang, N. *et al*. Hierarchical assembly of centriole subdistal appendages via centrosome binding proteins CCDC120 and CCDC68. *Nat. Commun.* **8**, 15057 doi: 10.1038/ncomms15057 (2017).

**Publisher's note:** Springer Nature remains neutral with regard to jurisdictional claims in published maps and institutional affiliations.

## Supplementary Material

Supplementary Information Supplementary Figures and Supplementary Tables
