# Supplementary material for: Hierarchical assembly of centriole subdistal appendages via centrosome binding proteins CCDC120 and CCDC68
Source: Nat Commun. 2017 Apr 19;8:15057. doi: 10.1038/ncomms15057 (PMC5399293; doi:10.1038/ncomms15057)
Supplement: Supplementary Information — Supplementary Figures and Supplementary Tables [file ncomms15057-s1.pdf]

Supplementary Figure 1

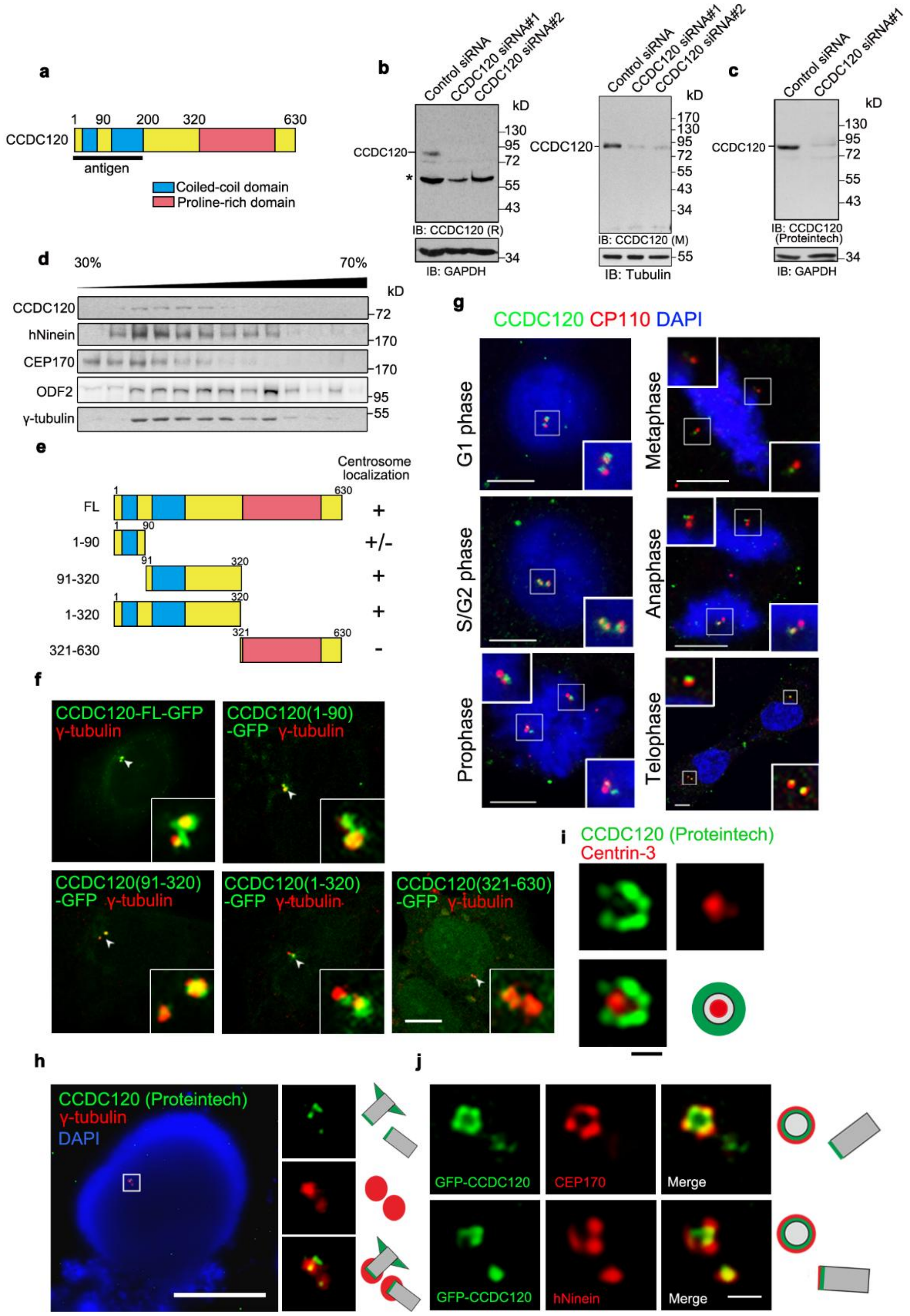

Supplementary Figure 1 CCDC120 is a centrosome protein. (**a**) Schematic of human CCDC120. Coiled-coil domains, blue; proline-rich domain, red; antigen, black line. (**b, c**) Immunoblot analysis of control- or CCDC120-siRNA-treated U2OS cell lysates with lab-generated rabbit (R), mouse (M) or commercial rabbit (Proteintech) polyclonal anti-CCDC120 antibody. GAPDH or tubulin was

used as a loading control. The asterisk marks a non-specific band. (**d**) Immunoblots showing that CCDC120-containing fractions are enriched with hNinein, CEP170, ODF2, and γ-tubulin. (**e**) Schematic of full-length (FL) CCDC120 and its truncates. Coiled-coil domains, yellow; proline-rich domains, red. Centrosome localization: ＋, positive; −, negative. (**f**) Immunostaining of γ-tubulin (red) in HeLa cells transfected with CCDC120-GFP truncates (green). The arrowheads indicate centrosome localization. Scale bar, 5 μm. (**g**) Immunostaining of CCDC120 (green) and CP110 (red) at different stages of the cell cycle in HeLa cells. DNA was stained with DAPI (blue). Scale bars, 5 μm. (**h**) Immunostaining of CCDC120 (green) and γ-tubulin (red) in U2OS cells. A commercial anti-CCDC120 antibody (Proteintech) was used. DNA was stained with DAPI (blue). Scale bar, 5 μm. (**i**) Immunostaining of CCDC120 (green) and Centrin-3 (red) in U2OS cells. Scale bar, 500 nm. (**j**) Immunostaining of CEP170 (red, top) or hNinein (red, bottom) in U2OS cells transfected with GFP-CCDC120 (green). Scale bar, 500 nm. Unprocessed original scans of immunoblots are shown in Supplementary Fig. 7.

Supplementary Figure 2

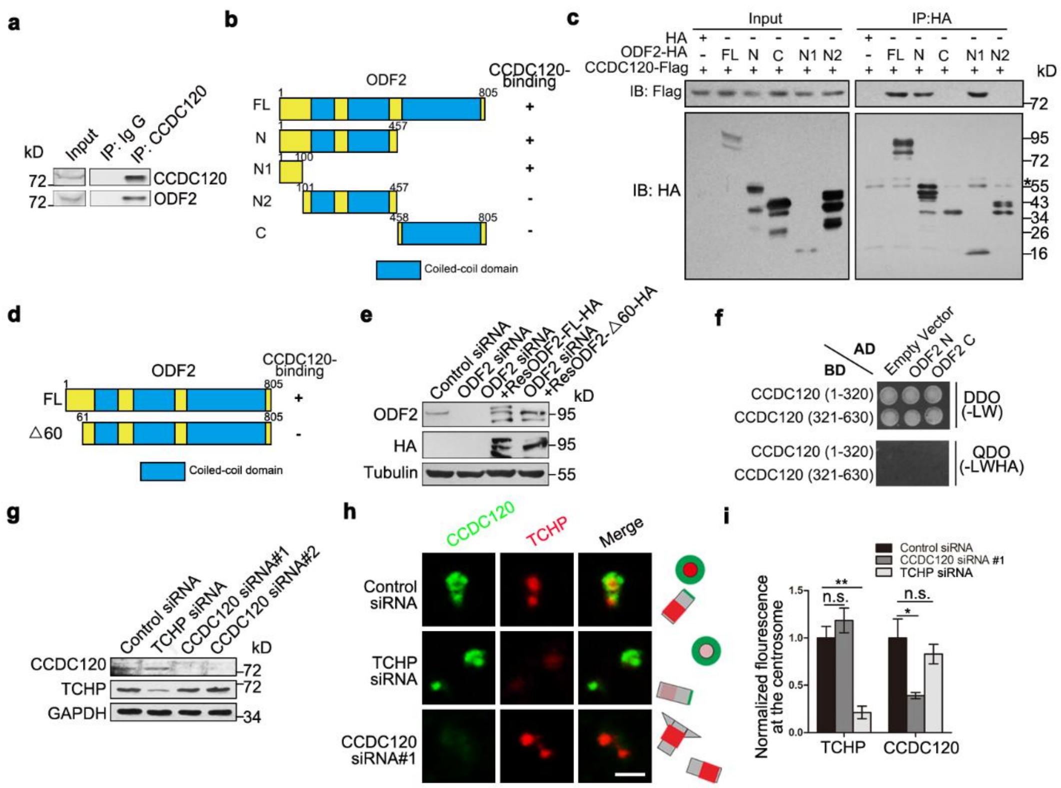

Supplementary Figure 2 ODF2, but not TCHP, recruits CCDC120 to the centrosome. (**a**) Immunoprecipitation (IP) of CCDC120 with ODF2 by anti-CCDC120 antibody (lab-generated, rabbit) in lysates of HeLa cells. (**b**) Schematic of full-length (FL) ODF2 and its truncates (N, N1, N2, C). Coiled-coil domains, blue. CCDC120 binding activity: ＋, positive; −, negative. (**c**) Lysates of HEK293T cells co-overexpressing CCDC120-Flag and the indicated ODF2-HA truncates were subjected to immunoprecipitation (IP) and immunoblotted (IB) with anti-Flag or anti-HA antibody. The asterisk marks IgG band. (**d**) Schematic of full-length (FL) ODF2 and its truncate lacking 1–60 aa (Δ60). Coiled-coil domains, blue. CCDC120 binding activity: ＋, positive; −, negative. (**e**) Immunoblots showing depletion of ODF2 by siRNA transfection and rescue of ODF2 expression by overexpressing HA-tagged siRNA-resistant full-length (ResODF2-FL) or the siRNA-resistant truncate lacking 1–60 aa (ResODF2-Δ60). Tubulin was used as a loading control. (**f**) Yeast two-hybrid assays. Interaction between CCDC120 truncates (1–320, 321–630 aa) and ODF2 truncates (N, 1–457 aa; C, 458–805 aa) was assessed. Transformed yeast cells were plated onto DDO media (−LW) and QDO media (−LWHA). AD, activation domain; BD, binding domain; DDO, double drop

out; QDO, quadruple drop out; LW, leucine, tryptophan; LWHA, leucine, tryptophan, histidine, and adenine. (**g**) Immunoblots showing depletion of TCHP or CCDC120 by siRNA in U2OS cells. GAPDH was used as a loading control. (**h**) Immunostaining of CCDC120 (green) and TCHP (red) in control-, TCHP-, or CCDC120-siRNA-transfected U2OS cells. Scale bar, 1 μm. (**i**) Quantification of the fluorescence intensity of TCHP and CCDC120 at centrosomes from **h** (n > 100 cells from three individual experiments). Data in **i** are the mean ± s. e. m. Statistical significance was determined by a two-sided Student's *t*-test. $^*P < 0.05$, $^{**}P < 0.01$; n.s., not significant. Unprocessed original scans of immunoblots are shown in Supplementary Fig. 7.

Supplementary Figure 3

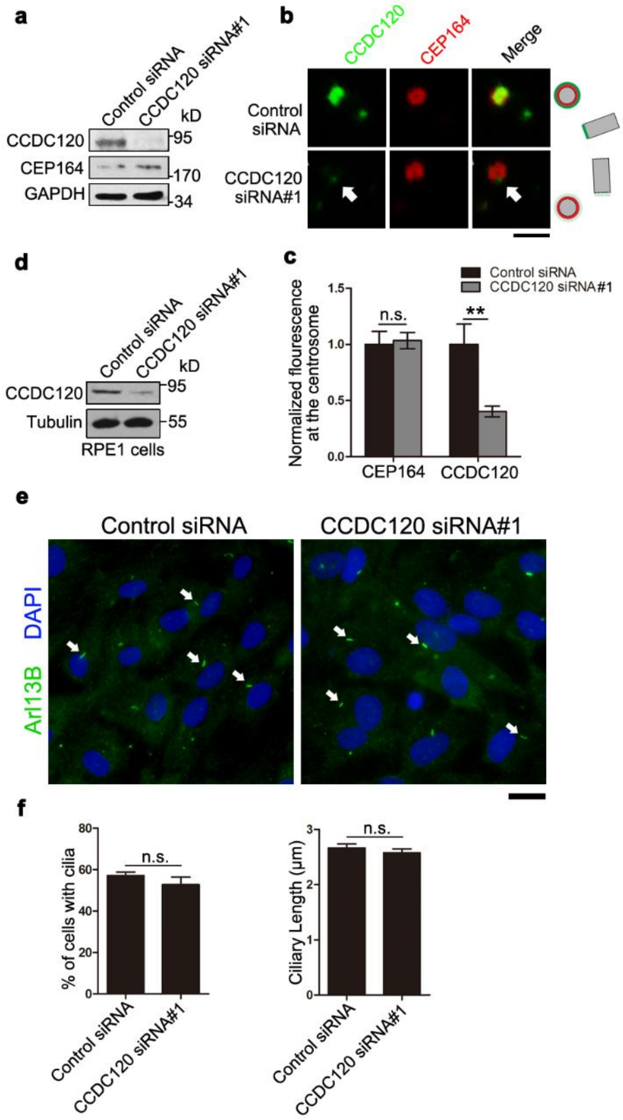

Supplementary Figure 3 CEP164 localization and cilia formation are not affected by CCDC120 depletion. (**a**) Immunoblots showing depletion of CCDC120 by siRNA in U2OS cells. GAPDH was used as a loading control. (**b**) Immunostaining of CCDC120 (green) and CEP164 (red) in control- or CCDC120-siRNA-transfected U2OS cells. The arrows indicate centrosome localization. Scale bar, 1 μm. (**c**) Quantification of the fluorescence intensity of CEP164 and CCDC120 at centrosomes from **b** ($n > 100$ cells from three individual experiments). (**d**) Immunoblots showing depletion of CCDC120 by siRNA in hTERT RPE-1 cells. Tubulin was used as a loading control. (**e**) Immunostaining of Arl13b (green) in control- or CCDC120-siRNA-

transfected hTERT RPE-1 cells. DNA was stained with DAPI (blue). The arrows indicate cilia localization. Scale bar, 5 μm. (**f**) Quantification of the number of cells with cilia and ciliary length (n > 100 cells from three individual experiments). hTERT RPE-1 cells were serum-starved for 48 h after transfection with the indicated siRNAs. Data in **c,** and **f** are the mean ± s.e.m. Statistical significance was determined by a two-sided Student's *t*-test. $^{**}P < 0.01$; n.s., not significant. Unprocessed original scans of immunoblots are shown in Supplementary Fig. 7.

Supplementary Figure 4

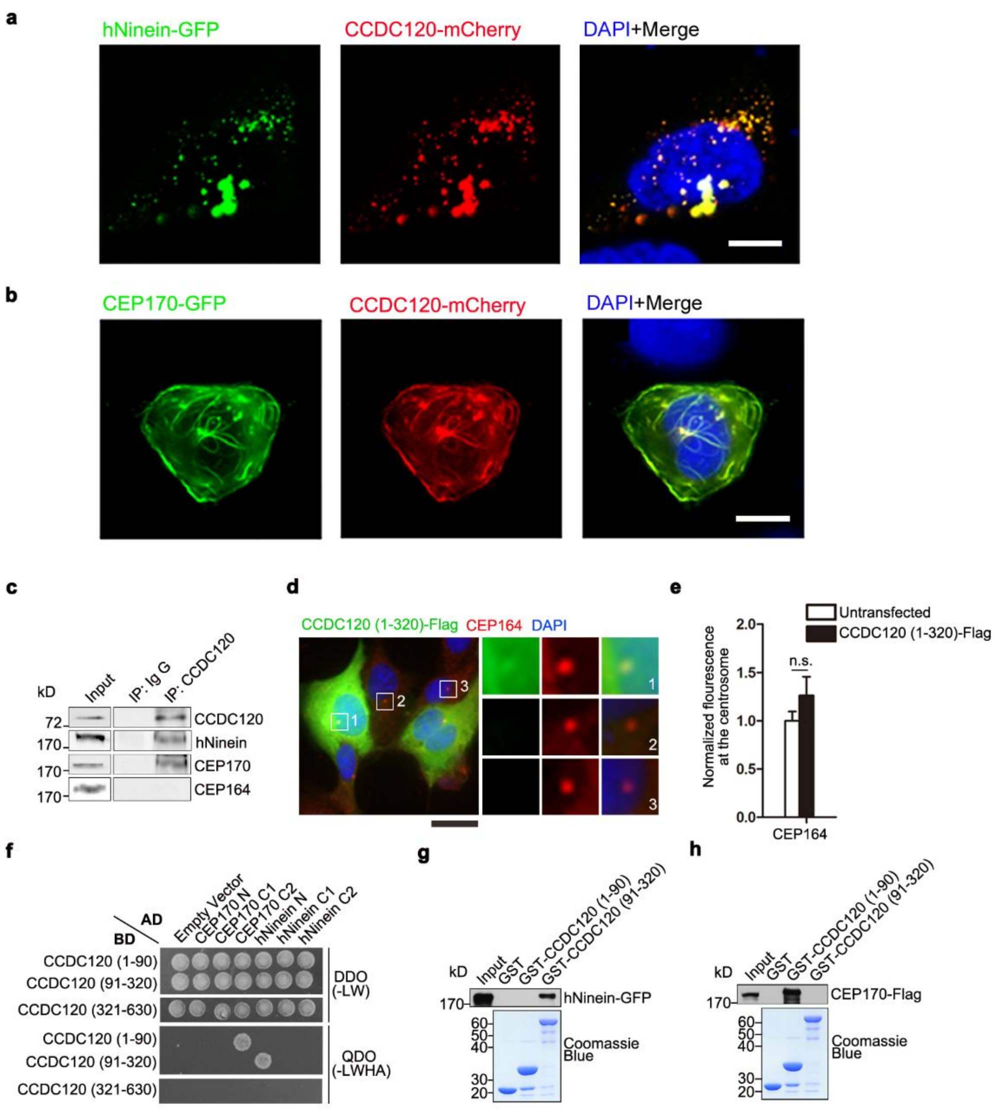

Supplementary Figure 4 CCDC120 interacts with hNinein and CEP170. (**a**) Confocal microscopy images of U2OS cells overexpressing hNinein-GFP (green) and CCDC120-mCherry (red). Scale bar, 5 μm. (**b**) Confocal microscopy images of U2OS cells overexpressing CEP170-GFP (green) and CCDC120-mCherry (red). Scale bar, 5 μm. (**c**) Immunoprecipitation (IP) of CCDC120 with hNinein, CEP170, and CEP164 by anti-CCDC120 antibody (lab-generated, rabbit) in HeLa cell lysates. (**d**) Immunostaining of Flag (green), and CEP164 (red) in U2OS cells overexpressing Flag-tagged CCDC120 (1–320 aa). DNA was stained with DAPI (blue). Centrosomes are magnified. Scale bar, 5 μm. (**e**) Quantification of the fluorescence intensity of CEP164 at centrosomes from **d** (n > 100 cells from three individual experiments). Data are the mean ±s. e. m. Statistical significance was determined by a two-sided Student's *t*-test. n.s., not significant.

(**f**) Yeast two-hybrid assays of CCDC120 truncates (1–90, 91–320 and 321–630 aa) with truncates of CEP170 (N, C1, C2) and hNinein (N, C1, C2). Transformed yeast cells were plated onto DDO media (−LW) and QDO media (−LWHA). AD, activation domain; BD, binding domain; DDO, double drop out; QDO, quadruple drop out; LW, leucine, tryptophan; LWHA, leucine, tryptophan, histidine, and adenine. (**g**, **h**) GST pull-down assays between CCDC120 and hNinein (**g**) or CCDC120 and CEP170 (**h**). Lysates of HEK293T cells overexpressing hNinein-GFP (**g**) or CEP170-Flag (**h**) were incubated with glutathione-agarose beads coated with GST, GST-CCDC120 (1–90 aa), or GST-CCDC120 (91–320 aa) and immunoblotted with anti-GFP (**g**) or anti-Flag (**h**) antibody. Unprocessed original scans of immunoblots are shown in Supplementary Fig. 7.

Supplementary Figure 5

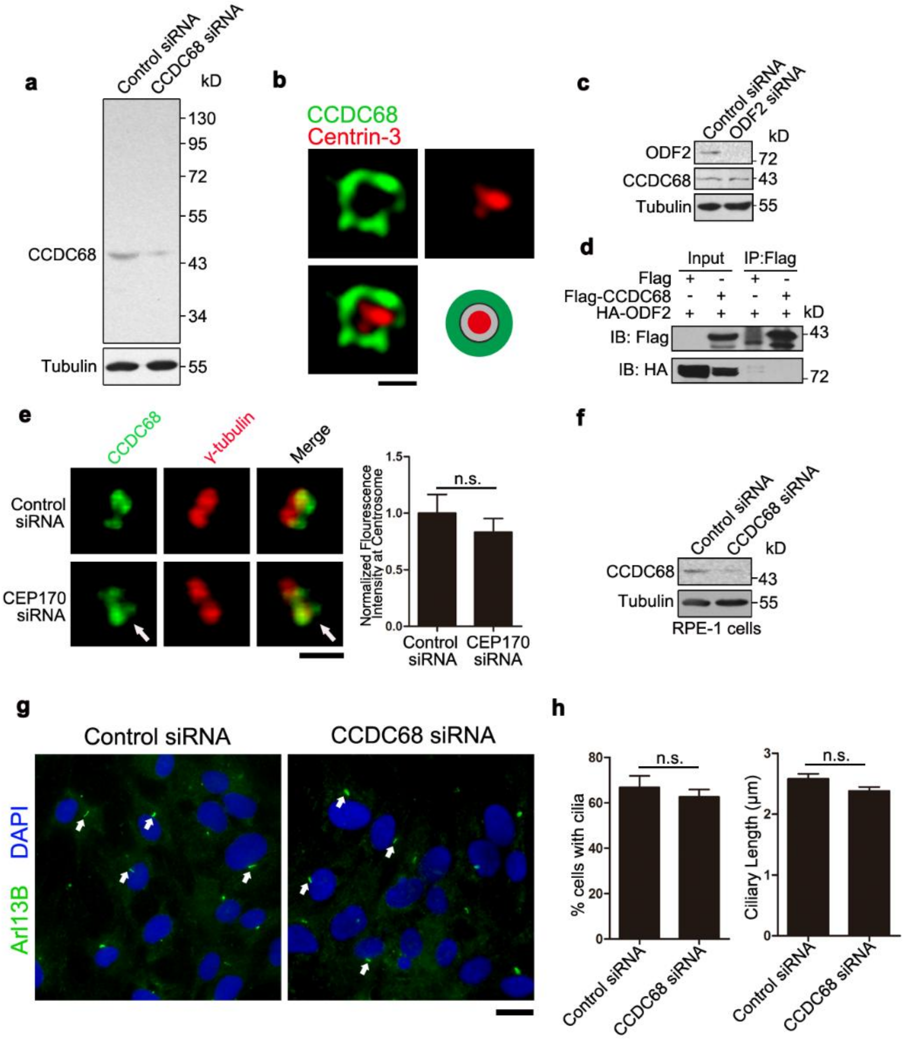

Supplementary Figure 5 ODF2 did not directly recruit CCDC68 to the SDAs, and CCDC68 depletion did not affect cilia formation in hTERT-RPE1 cells. (**a**) Immunoblot analysis of control- or CCDC68-siRNA-treated U2OS cell lysates with anti-CCDC120 antibody. Tubulin was used as a loading control. (**b**) Immunostaining of CCDC68 (green) and Centrin-3 (red) in U2OS cells. Scale bar, 500 nm. (**c**) Immunoblots showing depletion of ODF2 by siRNA in U2OS cells. Tubulin was used as a loading control. (**d**) Lysates of HEK293T cells co-overexpressing Flag-CCDC68 and HA-ODF2 were subjected to immunoprecipitation (IP) and immunoblotted (IB) with anti-HA or anti-Flag antibody. (**e**) Immunostaining of CCDC68 (green) and γ-tubulin (red) in control- or CEP170-siRNA-transfected U2OS cells. The arrows indicate centrosome localization. Scale bar, 1 μm. Quantification of the fluorescence intensity of CCDC68 at centrosomes are shown

at right (n > 100 cells from three individual experiments). (**f**) Immunoblots showing depletion of CCDC68 by siRNA in hTERT RPE-1 cells. Tubulin was used as the loading control. (**g**) Immunostaining of Arl13b (green) in control- or CCDC68-siRNA-transfected hTERT RPE-1 cells. DNA was stained with DAPI (blue). The arrows indicate cilia localization. Scale bar, 5 μm. (**h**) Quantification of the number of cells with cilia and ciliary length (n > 100 cells from three individual experiments). hTERT RPE-1 cells were serum-starved for 48 h after transfection with the indicated siRNAs. Data in **e** and **h** are the mean ± s.e.m. Statistical significance was determined by a two-sided Student's *t*-test. n.s., not significant. Unprocessed original scans of immunoblots are shown in Supplementary Fig. 7.

Supplementary Figure 6

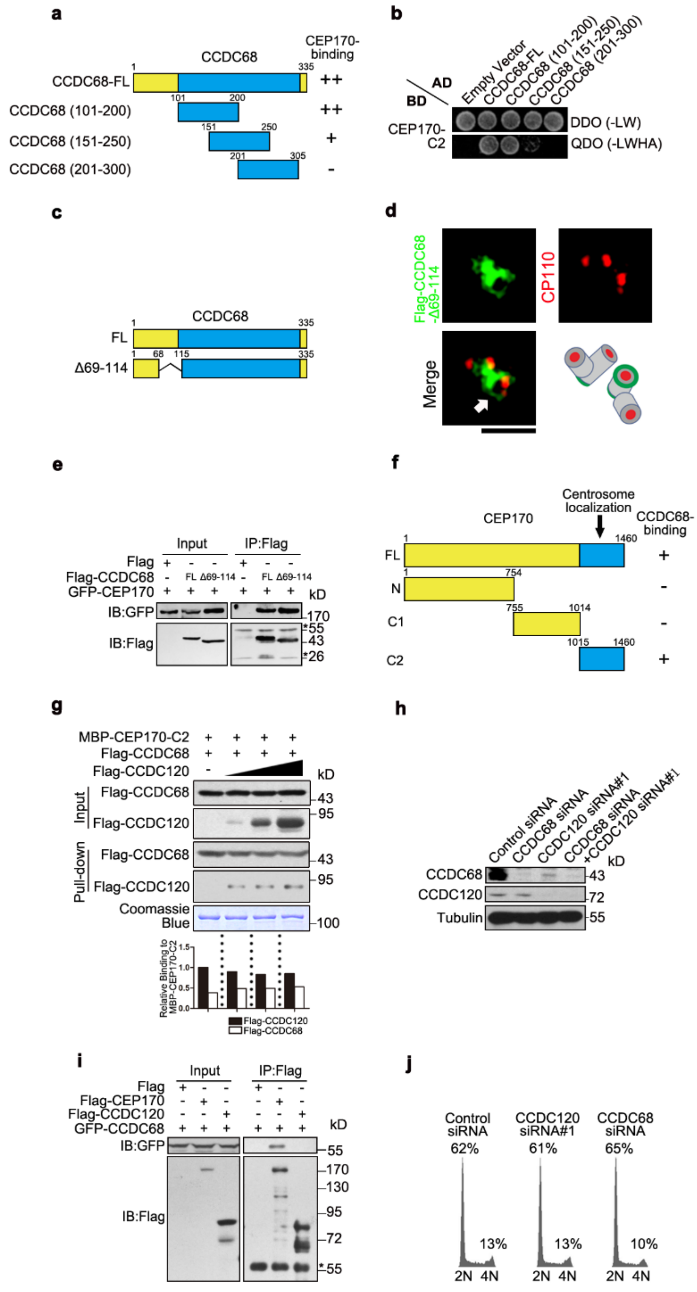

Supplementary Figure 6 The CCDC68 mutant lacking 69-114 aa showed no changes in CEP170 binding or SDA localization. (**a**) Schematic of full-length (FL) CCDC68 and its truncates. Coiled-coil domains, blue. CEP170 binding activity: ＋, positive; −, negative. (**b**) Yeast two-hybrid assays of full-length (FL) CCDC68 and its truncates (101–200, 151–250, and 201–300 aa) with the CEP170 truncate (C2, 1015–1460 aa). Transformed yeast cells were plated onto DDO media (−LW) and QDO media (−LWHA). AD, activation domain; BD, binding domain; DDO, double drop out; QDO, quadruple drop out; LW, leucine, tryptophan; LWHA, leucine, tryptophan, histidine, and

adenine. (**c**) Schematic of full-length (FL) CCDC68 and its truncate lacking 69–114 aa (CCDC68Δ69-114). Coiled-coil domains, blue. (**d**) Immunostaining of CP110 (red) and Flag (green) in Flag-CCDC68Δ69-114 overexpressing U2OS cells. The arrow indicates the ring-like structure formed by Flag-CCDC68Δ69-114 at the SDAs. Scale bar, 1 μm. (**e**) Lysates of HEK293T cells co-overexpressing GFP-CEP170 and full-length (FL) Flag-CCDC68 or mutant Flag-CCDC68 (Δ69-114) were subjected to immunoprecipitation (IP) and immunoblotted (IB) with anti-GFP and anti-Flag antibodies. The asterisks mark IgG bands. (**f**) Schematic of full-length (FL) CEP170 and its truncates (N, C1, C2). Coiled-coil domains are shown in blue. CCDC68 binding ability: ＋, positive; －, negative. (**g**) HEK293T cells overexpressing Flag-CCDC120 or Flag-CCDC68 were lysed and sequentially subjected to pull-down assays with MBP-CEP170-C2 (1015–1460 aa). Quantification of the Flag-CCDC68 and Flag-CCDC120 bands intensities in three independent experiments are shown. Both protein levels are normalized to that of MBP-CEP170-C2. (**h**) Immunoblots showing depletion of CCDC68, CCDC120, or CCDC68/CCDC120 by siRNA in U2OS cells. Tubulin was used as a loading control. (**i**) Lysates of HEK293T cells co-overexpressing GFP-CCDC68 and Flag-CEP170 or Flag-CCDC120 were subjected to immunoprecipitation (IP) and immunoblotted (IB) with anti-GFP and anti-Flag antibodies. The asterisk marks IgG band. (**j**) Flow cytometry analysis of control-, CCDC120-, and CCDC68-siRNA-treated HeLa cells. Unprocessed original scans of immunoblots are shown in Supplementary Fig. 7.

Supplementary Figure 7-1

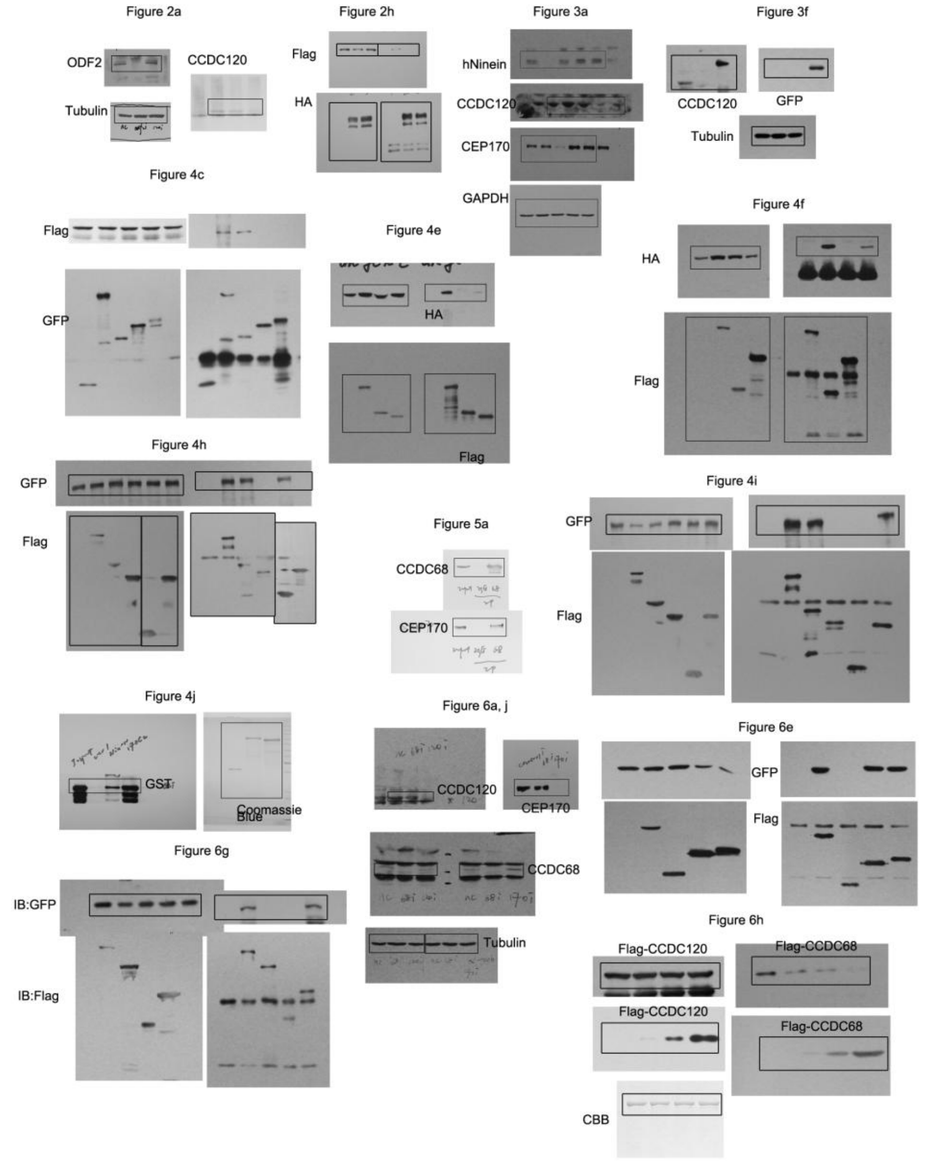

Supplementary Figure 7-2

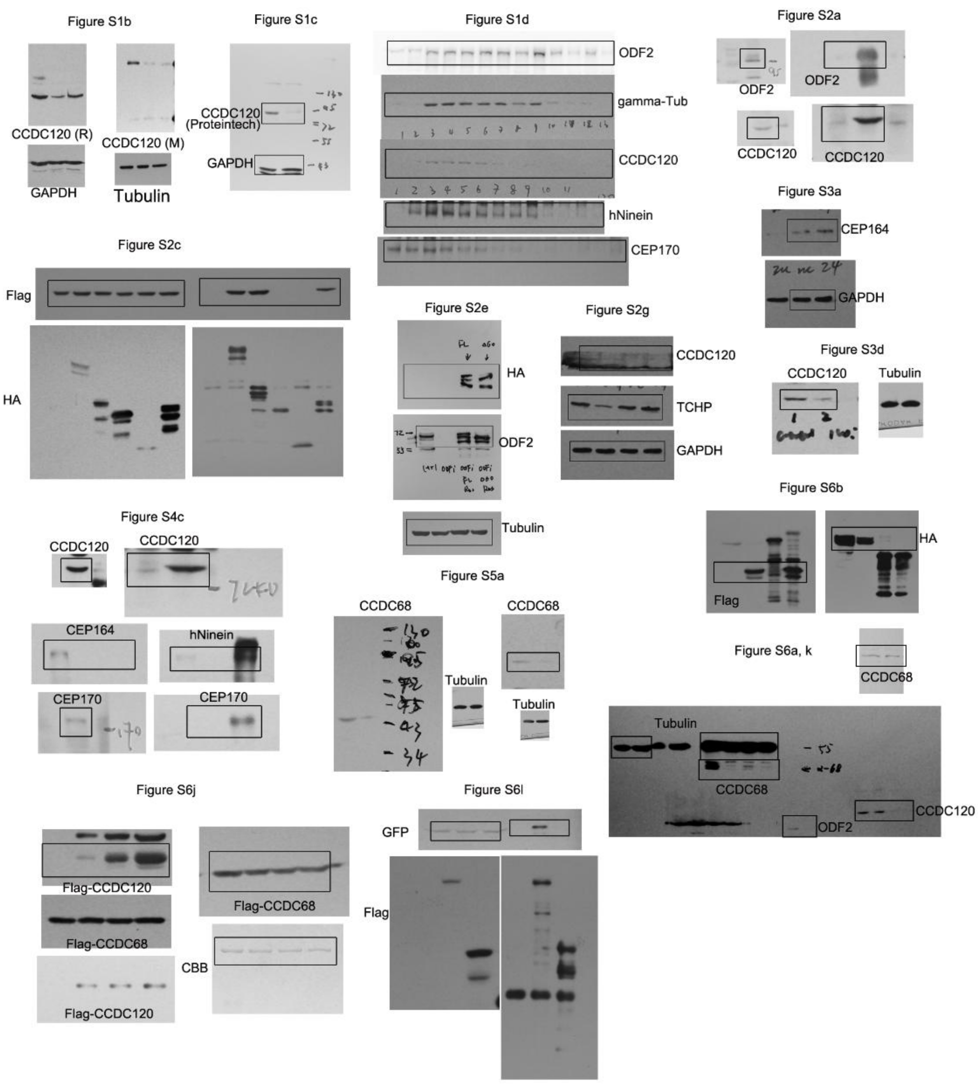

Supplementary Figure 7 Representative unprocessed scanned images of immunoblots.

Supplementary Table 1. A portion of CEP170-interacting proteins

Protein Name	Protein Description	Peptide Count	Sequence Coverage
CEP170	Centrosomal protein of 170 kDa OS=Homo sapiens GN=CEP170 PE=1 SV=1	21	15.7%
CEP170L	Cep170-like protein OS=Homo sapiens GN=CEP170P1 PE=5 SV=2	12	24.3%
CCDC68	Coiled-coil domain-containing protein 68 OS=Homo sapiens GN=CCDC68 PE=1 SV=1	10	19.7%
PCM-1	Pericentriolar material 1 protein OS=Homo sapiens GN=PCM1 PE=1 SV=4	7	8.7%
MARK2	Serine/threonine-protein kinase MARK2 OS=Homo sapiens GN=MARK2 PE=1 SV=2	6	10%
Nedd-1	Protein NEDD1 OS=Homo sapiens GN=NEDD1 PE=1 SV=1	4	4%
Ninein	Ninein OS=Homo sapiens GN=NIN PE=1 SV=4	3	4.2%
CCDC120	Coiled-coil domain-containing protein 120 OS=Homo sapiens GN=CCDC120 PE=1 SV=1	3	4.5%
KIF2A	Kinesin-like protein KIF2A OS=Homo sapiens GN=KIF2A PE=1 SV=3	1	3.1%
α-Tub	Tubulin alpha-1A chain OS=Homo sapiens GN=TUBA1A PE=1 SV=1	1	9.4%
RabGAP1	Rab GTPase-activating protein 1 OS=Homo sapiens GN=RABGAP1 PE=1 SV=3	1	1.4%

Supplementary Table 2. List of primary antibodies

Primary antibodies

Antigen	Source	Supplier	Cat. #	WB	IF
CCDC120 (1-200 aa)	Rabbit	Lab-generated		1:1000 Fig. 2a,3a,3d,6j. Supplementary Fig.1b,1d,2a,2g,3a,3c,4c,5f,6h.	1:100 Fig. 1a,2c,2f,5e,6k.
CCDC120 (1-200 aa)	Mouse	Lab-generated		1:1000 Supplementary Fig. 1b.	1:100 Fig. 1b-h,2b,2f,3b,3c. Supplementary Fig. 1g,2h,3b.
CCDC120 (281-630 aa)	Rabbit	Proteintech	22041-1-AP	1:500 Fig.2d,4a. Supplementary Fig.1c.	1:50 Supplementary Fig. 1h,1i.
Ninein	Rabbit	Dr. Mette M. Mogensen, University of East Anglia, UK		1:1000	1:200
hNinein (1-480 aa)	Rabbit	Lab-generated			1:200
CEP170	Rabbit	Abcam	ab72505	1:500	1:200
ODF2	Rabbit	Proteintech	12058-1-AP	1:1000	1:200
TCHP	Rabbit	Sigma-Aldrich	HPA038638	1:2000	1:200
CP110	Rabbit	Proteintech	12780-1-AP		1:200
CEP164	Rabbit	Proteintech	22227-1-AP	1:1000	1:200
Arl13b	Rabbit	Proteintech	17711-1-AP		
CCDC68	Rabbit	GeneTex	GTX106883	1:500	1:50
Centrin-3	Mouse	Abnova	3E6	1:500	1:200
C-Nap1	Mouse	Santa Cruz	F7		1:50
Cyclin B	Rabbit	Cell Signalling	#4138	1:500	
α-tubulin	Mouse	Sigma-Aldrich	DM1A	1:2000	
γ-tubulin	Rabbit	Sigma-Aldrich	T3559		1:200
γ-tubulin	Mouse	Sigma-Aldrich	GTU88	1:1000	1:200
GAPDH	Mouse	CWBIO	CW0110M	1:1000	
HA	Mouse	Sigma-Aldrich	HA-7	1:5000	1:1000
Flag	Mouse	Sigma-Aldrich	M2	1:1000	1:200
GFP	Rabbit	Lab-generated		1:5000	

Supplementary Table 3. List of secondary antibodies

Secondary antibodies						
**Name**	**Source**	**Label or Dye**	**Company**	**Cat. #**	**WB**	**IF**
Anti-Rabbit IgG (H+L)	Goat	HRP	Jackson ImmunoResearch	111-035-003	1:5000	
Anti-Mouse IgG (H+L)	Goat	HRP	Jackson ImmunoResearch	115-035-003	1:5000	
Anti-Rabbit IgG (H+L)	Goat	Alexa Flour 488	Invitrogen	A-11034		1:500
Anti-Mouse IgG (H+L)	Goat	Alexa Flour 488	Invitrogen	A-11039		1:500
Anti-Rabbit IgG (H+L)	Goat	Alexa Flour 568	Invitrogen	A-11036		1:500
Anti-Mouse IgG (H+L)	Goat	Alexa Flour 568	Invitrogen	A-11031		1:500
